# Three-Dimensional Printing and Tissue Engineering in Auricular Reconstruction: Global Trends, Research Frontiers, and a Translational Evidence Map

**DOI:** 10.3390/bioengineering13101095

**Published:** 2026-09-22

**Authors:** Ruohan Lv, Jingwei Feng, Yuhui He, Yanyong Zhao

**Affiliations:** 1 Plastic Surgery Hospital and Institute, Chinese Academy of Medical Sciences and Peking Union Medical College, Beijing 100144, China; 2 China-Japan Friendship Hospital, Beijing 100029, China

**Keywords:** auricular reconstruction, three-dimensional printing, tissue engineering, bibliometric analysis, science mapping, evidence map, translational maturity, cartilage regeneration

## Abstract

Three-dimensional (3D) printing and tissue engineering offer patient-specific strategies for auricular reconstruction, but bibliometric structure alone cannot establish technical maturity or clinical value. We combined bibliometric mapping of English-language Articles and Reviews in the Web of Science Core Collection (2014–2025) with a translational evidence map. The locked corpus comprised 169 publications (143 Articles and 26 Reviews) and 4291 citations at the 12 June 2026 snapshot. Annual output, countries, institutions, authors, journals, highly cited records, author-keyword co-occurrence, and temporal terminology were analyzed. Citation-age sensitivity used citations per publication-year (CPY), year-normalized scores, and within-year percentiles. Record-level coding evaluated technical routes, materials, cell systems, construct scale, evidence stage, scientific endpoints, reporting completeness, and barriers. Among Articles, 40 (28.0%) were in vitro only, 41 (28.7%) reached small-animal testing, four (2.8%) reached large-animal testing, and nine (6.3%) reported human evidence; only one was comparative clinical research. Chondrogenesis or extracellular-matrix outcomes were reported in 84 Articles (58.7%), whereas vascularization, clinical/aesthetic outcomes, and long-term safety/adverse events were each reported in ≤6.3%. The field is expanding internationally but remains predominantly preclinical; progress requires standardized reporting, native-cartilage comparators, long-term shape and safety assessment, and larger-animal and comparative clinical validation.

## 1. Introduction

Auricular reconstruction is among the most technically demanding procedures in plastic and reconstructive surgery because the external ear combines a complex three-dimensional (3D) geometry with thin soft-tissue coverage and distinctive elastic-cartilage mechanics [1]. Established approaches, principally autologous costal cartilage frameworks and porous polyethylene implants, can achieve durable reconstruction [2]. Nevertheless, they remain limited by donor-site morbidity, multistage procedures, demanding framework fabrication, constrained cartilage availability in young patients, and risks of exposure, infection, distortion, or extrusion [3]. These limitations have sustained interest in patient-specific engineering strategies capable of reproducing both auricular anatomy and the biological characteristics of elastic cartilage.

Global procedure registries and Global Burden of Disease (GBD) outputs do not disaggregate the number of auricular reconstruction procedures or microtia/anotia-specific disability-adjusted life years (DALYs). Nevertheless, the condition lies within a substantial congenital-anomaly burden: a GBD 2021-based analysis estimated 7.2 million congenital-birth-defect cases and 0.53 million associated deaths worldwide in 2021, and congenital birth defects ranked third among DALY causes in people younger than 20 years [4]. Population-based reports cite approximately one external-ear malformation per 6000–6830 newborns; depending on laterality and associated canal anomalies, these conditions may involve conductive hearing loss and psychosocial burden [5].

The convergence of 3D printing, additive manufacturing, biomaterials science, and tissue engineering has expanded the range of possible solutions. Image-based manufacturing can reproduce patient-specific anatomy, while extrusion bioprinting and related biofabrication methods permit spatial control over cells, hydrogels, polymers, extracellular-matrix components, and bioactive factors [6,7]. Recent work has investigated reinforced hydrogel constructs, polycaprolactone frameworks, decellularized cartilage matrices, chondrocyte-containing bioinks, perichondrium–cartilage integration, and strategies for vascularization and long-term shape retention [6,7,8]. This research addresses a clinically important condition: microtia and anotia occur worldwide, with prevalence varying across populations and regions [9].

These advantages must be balanced against platform-specific process limitations. In extrusion-based bioprinting, high-viscosity or cell- and particle-laden formulations may require pressures that increase nozzle shear exposure and the risk of clogging, whereas droplet-based approaches operate within narrower viscosity and cell-loading ranges and can generate satellite droplets that reduce placement fidelity [10,11].

Although narrative and systematic reviews have summarized individual technologies, they do not fully describe how the research landscape is distributed across years, countries, institutions, authors, and journals, or how recurring concepts relate to one another. Bibliometric analysis can complement technical reviews by quantifying publication patterns and mapping relationships among bibliographic entities and author-supplied terminology [12,13]. Its interpretive value, however, depends on a clearly delimited corpus, reproducible data transformations, transparent counting rules, and caution when citation or keyword indicators are used as proxies for scientific importance.

A complete landscape map remains useful, but publication growth, national output, raw citations, and keyword frequency are not direct measures of experimental validity, technical superiority, or clinical readiness. Citation counts are strongly affected by exposure time, and keyword networks cannot determine whether a platform has progressed from material characterization to animal validation or human reconstruction. A record-level content map is therefore needed to supplement—not replace—the bibliometric analysis by connecting publication structure with materials, cells, construct scale, scientific endpoints, evidence maturity, and explicitly reported translational barriers.

This study integrates a bibliometric analysis of 3D printing and tissue engineering for auricular reconstruction published from 2014 to 2025 with a structured translational evidence map. The objectives were to: (1) characterize annual publication output and fixed-snapshot citation patterns; (2) describe countries/regions, institutions, authors, journals, and highly cited studies; (3) construct a reproducible author-keyword co-occurrence network and evaluate descriptive temporal changes in terminology; (4) assess citation-age sensitivity using CPY, Y-NCS, and within-year percentiles; (5) classify technical routes, material and cell systems, construct scale, and highest evidence stage; and (6) quantify scientific endpoints, reporting completeness, and explicitly stated barriers to translation. Record-level eligibility decisions, bibliometric memberships, keyword transformations, content codes, source-linked evidence, validation outputs, full analysis and figure-generation code, version-pinned dependencies, run metadata, checksums, and figure-source tables accompany the article.

## 2. Materials and Methods

### 2.1. Study Design and Data Source

A structured bibliometric design was used to examine publications on 3D printing and tissue engineering in auricular reconstruction. The literature was retrieved from the Web of Science Core Collection (WoSCC), selected for its standardized document-type classification and consistent cited-by field. The database search and Cited_By_Count snapshot were recorded on 12 June 2026, and supplementary metadata enrichment was completed on 13 July 2026. Analytical files were locked on 6 September 2026. Only the WoSCC Cited_By_Count field was used in citation analyses; CrossRef citation values were retained as auxiliary metadata but were not combined with the WoSCC snapshot.

### 2.2. Search Strategy

The topic search combined a fabrication and biofabrication concept block with an auricular anatomy and pathology block:

TS = (“3D printing” OR “three-dimensional printing” OR “bioprinting” OR “additive manufacturing” OR “tissue engineering” OR “scaffold*” OR “biofabrication” OR “biomaterial*”) AND TS = (“auricular reconstruction” OR “ear reconstruction” OR “microtia” OR “auricular cartilage” OR “ear cartilage” OR “pinna” OR “auricle” OR “external ear”).

The publication window was 1 January 2014 to 31 December 2025. The lower bound was chosen to capture the period in which extrusion-based bioprinting and patient-specific fabrication of ear-shaped cartilage constructs emerged as a sustained research direction. The complete syntax, filtering sequence, and counts at each stage are provided in Supplementary Information S1.

### 2.3. Eligibility Criteria and Study Selection

Eligible records were English-language, full-length journal Articles or Reviews published during 2014–2025. They were required to directly investigate or substantively synthesize 3D printing, bioprinting, additive manufacturing, biomaterials, scaffold fabrication, biofabrication, or tissue engineering for reconstruction of the external auricle/pinna or regeneration or replacement of auricular elastic cartilage. Empirical studies had to fabricate, test, implant, or clinically apply an auricular-directed reconstructive scaffold, biomaterial, biofabricated construct, or tissue-engineered strategy. Reviews required a substantive focus on auricular bioengineering.

Records were excluded if they addressed developmental biology, molecular mechanisms, cell characterization, native-cartilage characterization, or biologic injection without a qualifying engineered construct. Further exclusions comprised non-auricular targets that merely used auricular cells or tissue; radiotherapy boluses; hearing devices; external prostheses or epitheses; splints, retainers, and orthoses; surgical planning, imaging, simulation, training, temporary templates, or anatomical models without an implanted or biologically evaluated reconstructive construct; conventional surgery without a qualifying engineered component; in silico designs without fabrication or biological/clinical evaluation; conference abstracts, theses, correspondence, and other non-full-length publication forms; retracted records; and records for which available evidence was insufficient to establish eligibility.

The initial topic search identified 1542 records. Database restrictions on publication year, document type, and language reduced this set to 1253 records. Sequential relevance and scope screening retained 569, 386, 310, 294 records. A final complete record-level eligibility audit of the 294 candidate inclusions applied the direct-application criteria above and excluded 125 records, yielding 169 publications (Figure 1).

**Figure 1:**
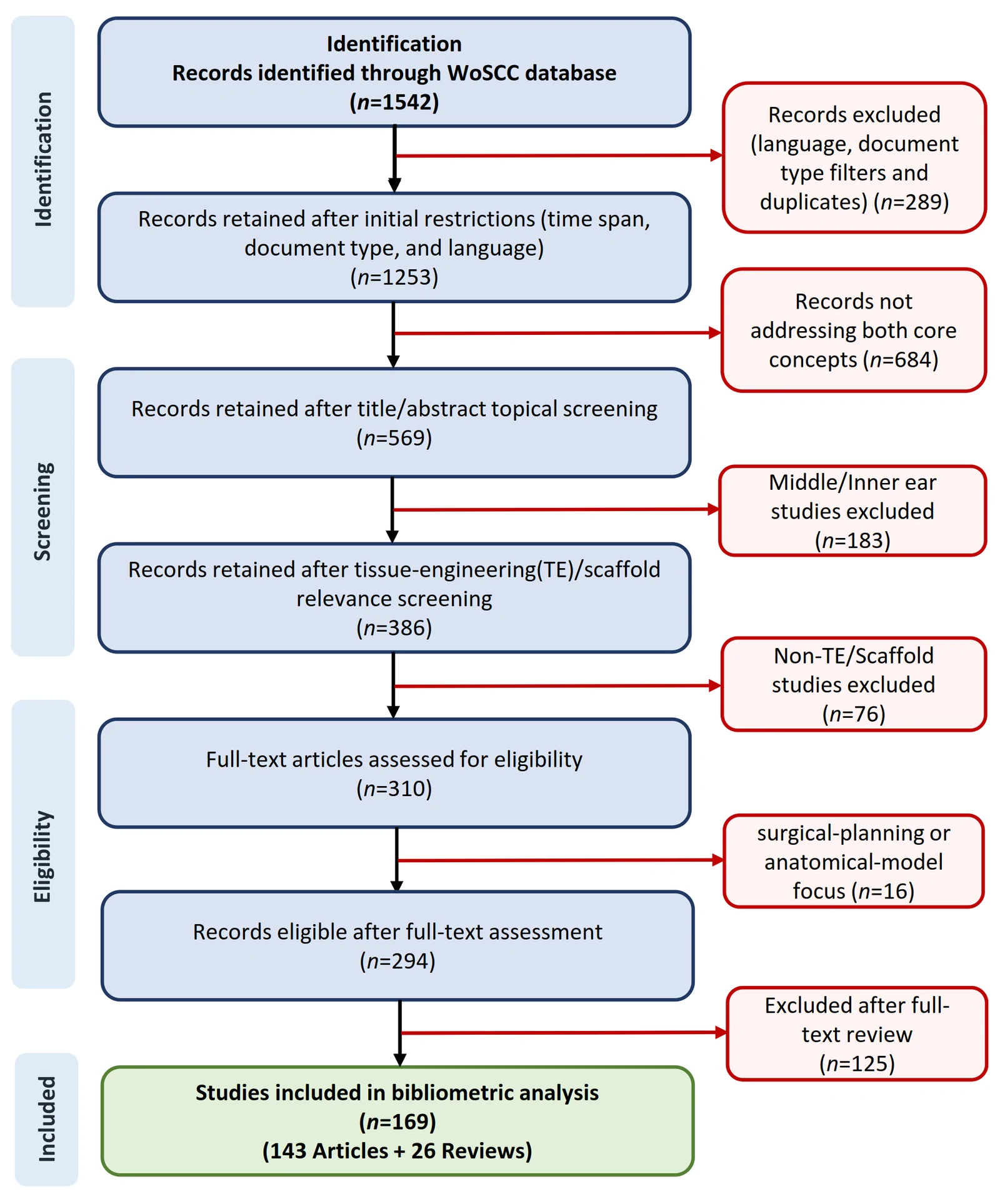
Flow diagram of the literature identification, screening, eligibility assessment, and final inclusion. The documented selection stages yielded 264 candidate inclusions; a complete record-level eligibility audit excluded 95 records and produced a final corpus of 169 full-length Articles and Reviews. Stable Record_ID values link the released 294-record late-stage decision log.

Each record in the released late-stage set was assigned an immutable identifier (Record ID = AR- plus a zero-padded original record number), final decision, reason family, rationale, confidence level, and evidence source. The released decision log covers all 169 candidate inclusions and previously documented exclusions from the 294-record late-stage set.

### 2.4. Data Extraction and Standardization

For each included publication, the master dataset preserved the title, full author list, standardized journal title, WoS publication year, document type, digital object identifier or persistent link, affiliation string, author keywords, abstract, and fixed-snapshot citation count. Abstracts were available for 146 of 169 records (86.4%), author keywords for 91 (53.8%), and affiliation strings for 140 (82.8%). The WoS publication-year field was used for all annual analyses; later formal issue years shown in reference lists for early-online articles were not substituted into the annual series.

Cited_By_Count was available for all retained records and yielded a total of 4291 citations. To reduce publication-age bias, each record received a year-normalized citation score (Y-NCS), calculated as the record’s citation count divided by the mean citation count of all final-corpus records from the same publication year. For an entity, the mean normalized citation score (MNCS) was the arithmetic mean of its constituent Y-NCS values. A tie-aware within-year percentile was calculated as 100 × (L + 0.5E)/N, where L is the number of same-year records with fewer citations, E is the number with the same citation count, and N is the number of final-corpus records in that year. These measures are internal age adjustments and should not be interpreted as external field- or category-normalized impact indicators.

Country and institution labels were standardized from affiliation strings and released as record-to-entity membership tables. Country analysis used whole counting, such that every listed country received one publication credit for a multinational record. Institutions were retained at the explicit organization level stated in the affiliation; documented aliases were consolidated, whereas generic department or school labels were excluded. Authors were parsed from the complete author field and counted once per record. Journal titles were standardized case-insensitively. Rankings were ordered by publication count, with total citations used only to break ties. Normalized citation measures were presented as descriptive companion metrics and were not used to infer research quality, leadership, or causal venue effects.

### 2.5. Bibliometric Analysis

All analyses were computed from the locked 169-record master table using the accompanying analysis scripts. Supplementary Figure Data contains the exact source rows used for all figures. 

#### 2.5.1. Publication and Citation Indicators

Descriptive statistics included annual publication and cumulative output, annual total, mean, and median citations, and the proportion of uncited records. Endpoint compound annual growth rate (CAGR) was calculated as [(publications in 2025/publications in 2014)^(1/11) − 1] × 100. Raw citation totals and means were treated as fixed-snapshot descriptions, with explicit recognition that newer records had less time to accumulate citations.

#### 2.5.2. Entity-Level Analyses

Countries/regions, institutions, authors, and journals were summarized by whole-counted publication frequency. For each entity, the analysis reported total raw citations, MNCS, mean within-year percentile, and the percentage of records at or above the 75th within-year percentile. Country percentages used the 169-record corpus as the denominator and may sum to more than 100% because of whole counting. Complete record-level membership tables support regeneration of every aggregate.

#### 2.5.3. Author-Keyword Network and Temporal Analysis

Author keywords were decoded for HTML entities and split on semicolons. Unicode, case, punctuation, whitespace, unambiguous spelling variants, singular/plural variants, and a limited set of abbreviations were standardized using a controlled Keyword_Synonym_Map. Broad conceptual merging was not applied. Repeated normalized terms within a record were counted once. The 91 records with usable author keywords contributed 470 normalized record-term occurrences. The analytical term set comprised 50 keywords occurring in at least two records, representing 255 occurrences within the thresholded set.

Co-occurrence was defined as the number of records in which two normalized terms appeared together. The complete symmetric matrix included all 50 terms. The displayed network retained undirected edges with co-occurrence weight of at least two, leaving 29 non-isolated plotted nodes and 58 weighted edges. Communities were identified with the NetworkX 3.6.1 implementation of the Louvain algorithm using co-occurrence weight, resolution 1.0, convergence threshold 1 × 10^−7^, and random seed 42 [14,15]. Coordinates were generated using a weighted spring layout with seed 42 and 500 iterations. Node area represented record frequency, edge width represented co-occurrence weight, and node color represented community. Terms isolated by the edge threshold remained in the complete matrix and node table but were not displayed in the network.

Temporal change was calculated for each of the 50 terms with frequency of at least two. Let n1(k) and n2(k) denote the number of records containing term k in 2014–2019 and 2020–2025, respectively. The descriptive trend index was TI(k) = n2(k)/336 − n1(k)/134, where 336 and 134 are the total normalized, within-record-deduplicated author-keyword occurrences in the two periods. The calculation table reports both numerators, denominators, period-specific shares, first and last occurrence years, and the resulting difference. The index is a descriptive share difference rather than a significance test or burst statistic.

#### 2.5.4. Highly Cited Records

The ten records with the highest raw Cited_By_Count values were tabulated with their Y-NCS and within-year percentile. This analysis describes citation concentration at the fixed snapshot; it does not identify an intellectual core or establish scientific quality, leadership, or causal influence.

#### 2.5.5. Citation-Age Sensitivity Analysis

To address unequal citation windows, citations per publication-year (CPY) were calculated as Cited_By_Count/max(1, 2026 − publication year). This simple annual-resolution sensitivity measure cannot account for the exact online publication date. CPY was therefore reported alongside the pre-existing Y-NCS and tie-aware within-year percentile. None of these indicators were interpreted as scientific quality, technical superiority, or clinical value.

### 2.6. Structured Content Coding and Translational Evidence Mapping

A prespecified codebook was applied to each record’s title, abstract, author keywords, and document type. The unit of analysis was the individual Record_ID. Reviews and Articles were analyzed separately for evidence maturity. For Articles, the highest identifiable evidence stage was coded as methods/modeling only, ex vivo only, in vitro only, small-animal in vivo, large-animal in vivo, human clinical, or not reported. Clinical translation level ranged from basic/methods to comparative clinical research and was required to agree with the highest evidence stage.

Construction strategies were multi-label and included conventional seeded scaffolds, 3D-printed acellular frameworks, 3D-printed cell-seeded scaffolds, cell-laden bioprinting, mold/cast constructs, decellularized native scaffolds, scaffold-free methods, and hybrid/composite systems. Material families included synthetic polymers, natural hydrogels or polymers, decellularized extracellular matrix or tissue, cellulose, silk, reinforced composites, alloplastic clinical implants, and cell-only or scaffold-free approaches. Cell strategies distinguished auricular or microtia chondrocytes, other chondrocytes, mesenchymal or bone-marrow stromal cells, adipose-derived stromal cells, other stem/progenitor cells, mixed-cell or co-culture systems, and cell-free constructs.

Scientific endpoints were coded only when the title or abstract explicitly stated that they were evaluated. These endpoints included cell viability/proliferation, chondrogenesis or extracellular-matrix formation, elastin or elastic-cartilage phenotype, mechanical properties, shape fidelity, post-implantation shape retention or contraction, degradation, inflammation or biocompatibility, vascularization or perfusion, host integration or skin coverage, clinical/aesthetic outcomes, and long-term safety or adverse events. Reporting fields recorded whether the abstract stated sample size, follow-up duration, mechanical testing, histology or matrix assays, a native-cartilage comparator, or adverse events.

Barriers were coded only when the title or abstract explicitly described a limitation, challenge, unresolved problem, or design objective. Prespecified categories were cell supply or dedifferentiation, mechanical instability, shape loss or shrinkage, vascularization or nutrition, degradation mismatch, inflammation or immunogenicity, skin or soft-tissue coverage, manufacturing reproducibility, limited long-term evidence, and regulatory or clinical-workflow barriers. Absence of a code indicates that the item was not explicit in the available record; it does not prove that the study did not assess it.

### 2.7. Coding Validation and Statistical Summaries

Content coding used a two-pass computer-assisted workflow with source-linked evidence. Every record retained an evidence quotation and coding-confidence label. A second independent pass was triggered for 136 records with low or moderate confidence, advanced evidence stage, positive barrier coding, or a validation flag; 33 low-risk records retained first-pass coding. The merged 169-record table underwent deterministic checks for unique identifiers, document-type logic, controlled vocabulary, evidence-stage/translation-level agreement, information-basis consistency, paired mechanical-test fields, and verbatim evidence. The final validation report contained zero errors and zero warnings.

Counts and percentages were descriptive. Article-level percentages used 143 Articles as the denominator unless stated otherwise. Because content fields were multi-label, strategy, material, cell, endpoint, and barrier percentages may sum to more than 100%. Records with insufficient information were retained as NR rather than imputed. The content map is conservative and represents title/abstract-level evidence visibility; it is not a replacement for full-text risk-of-bias assessment or meta-analysis.

## 3. Results

### 3.1. Corpus and Study Selection

The final corpus contained 169 publications, including 143 Articles (84.6%) and 26 Reviews (15.4%) (Figure 1). At the 12 June 2026 snapshot, the corpus had accumulated 4291 WoSCC citations, with a mean of 25.39 citations per publication. The dataset contained 27 country/region labels, 386 released institution labels, 959 author labels, of which 162 appeared in at least two publications, and 103 standardized journal titles. Country mappings were available for 139 records, institution mappings for 140, and author-keyword analysis for 91; the applicable denominator is reported for each analysis.

### 3.2. Publication and Citation Trends

Annual output increased from 10 publications in 2014 to 20 in 2025, corresponding to an endpoint CAGR of 6.5% across 11 annual intervals (Figure 2). Growth was not monotonic: output fell to five in 2017, increased to 17 in 2021, declined to 14 in 2022, and reached 20 in both 2023 and 2025. Eighty-eight publications (52.1%) were issued during 2021–2025.

**Figure 2:**
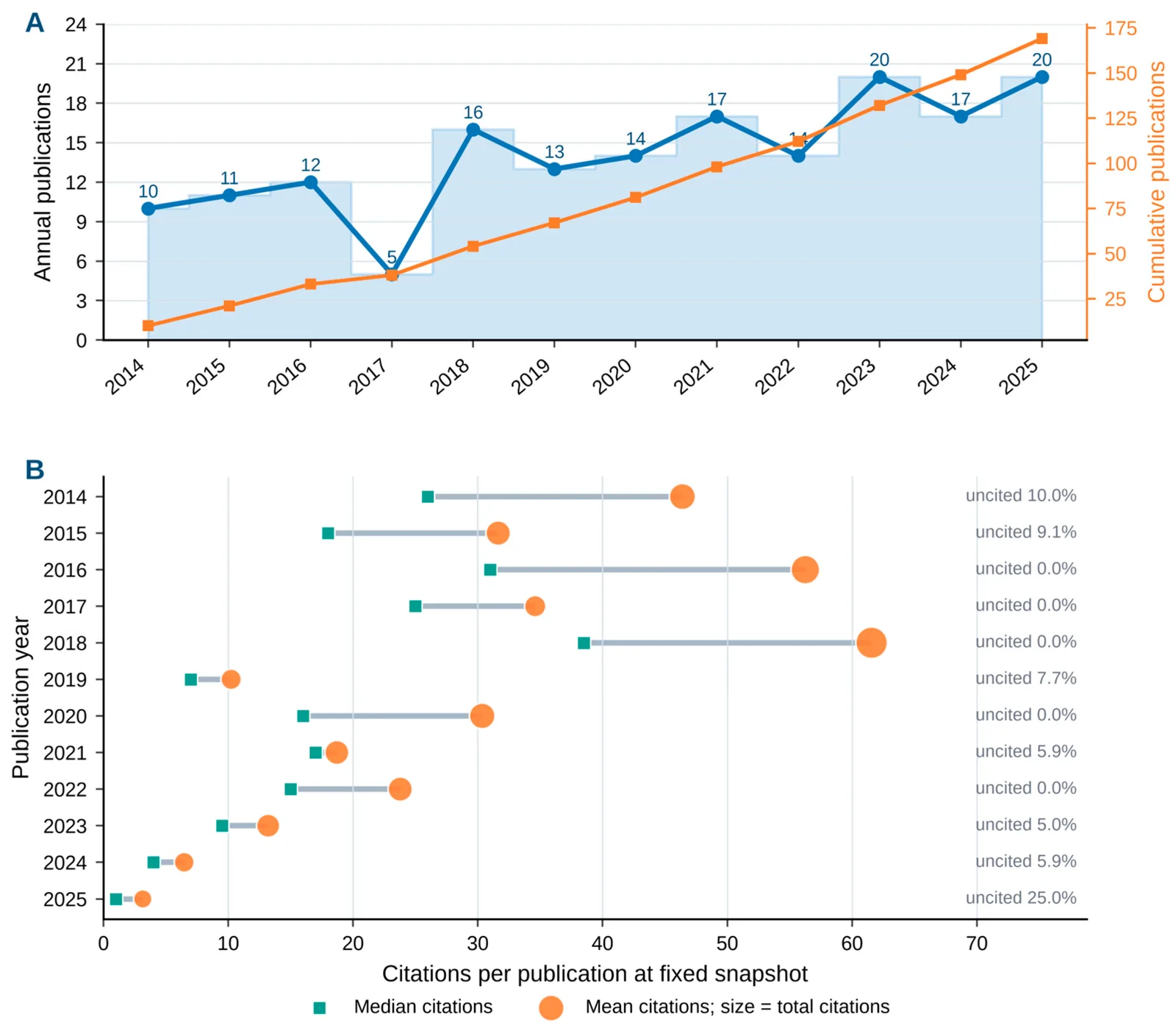
Annual publication activity and fixed-snapshot citation profile. (**A**) displays annual and cumulative publication output from 2014 to 2025. (**B**) Horizontal segments connect the median (squares) and mean (circles) WoSCC citations for each publication year; circle area represents the annual total citation count, and right-hand labels give the proportion of uncited records. Recent publication cohorts have had less time to accrue citations.

Raw citation accumulation was strongly associated with publication year. The 2018 cohort had the largest total (985) and mean (61.56) citation count, followed by the 2016 cohort (675 total; mean 56.25). The 2024 and 2025 cohorts averaged 6.47 and 3.15 citations, respectively, and five of the 20 records published in 2025 were uncited at the snapshot. These differences reflect unequal citation windows; cross-year comparisons therefore emphasize within-year normalization rather than raw means.

### 3.3. Global Contributions

Twenty-seven country/region labels occurred in the corpus, and 139 records had usable country mappings. China had the largest whole-counted output with 42 country-attributed publications (24.9% of the corpus), followed by the United States with 40 (23.7%). The Netherlands and Japan each contributed 12 publications (7.1%), while the United Kingdom and South Korea each contributed 11 (6.5%) (Table 1; Figure 3). Because whole counting credits every listed country on a multinational paper, country-attributed totals are not mutually exclusive.

Age-normalized citation summaries did not mirror the output ranking. Among countries with at least 10 publications, MNCS was 1.59 for The Netherlands, 1.29 for China, 1.18 for Republic of Korea, 1.10 for the United Kingdom, 0.97 for the United States, and 0.44 for Japan. These values adjust only for publication year within this corpus and do not control for topic, document type, team size, or field-wide citation expectations.

**Figure 3:**
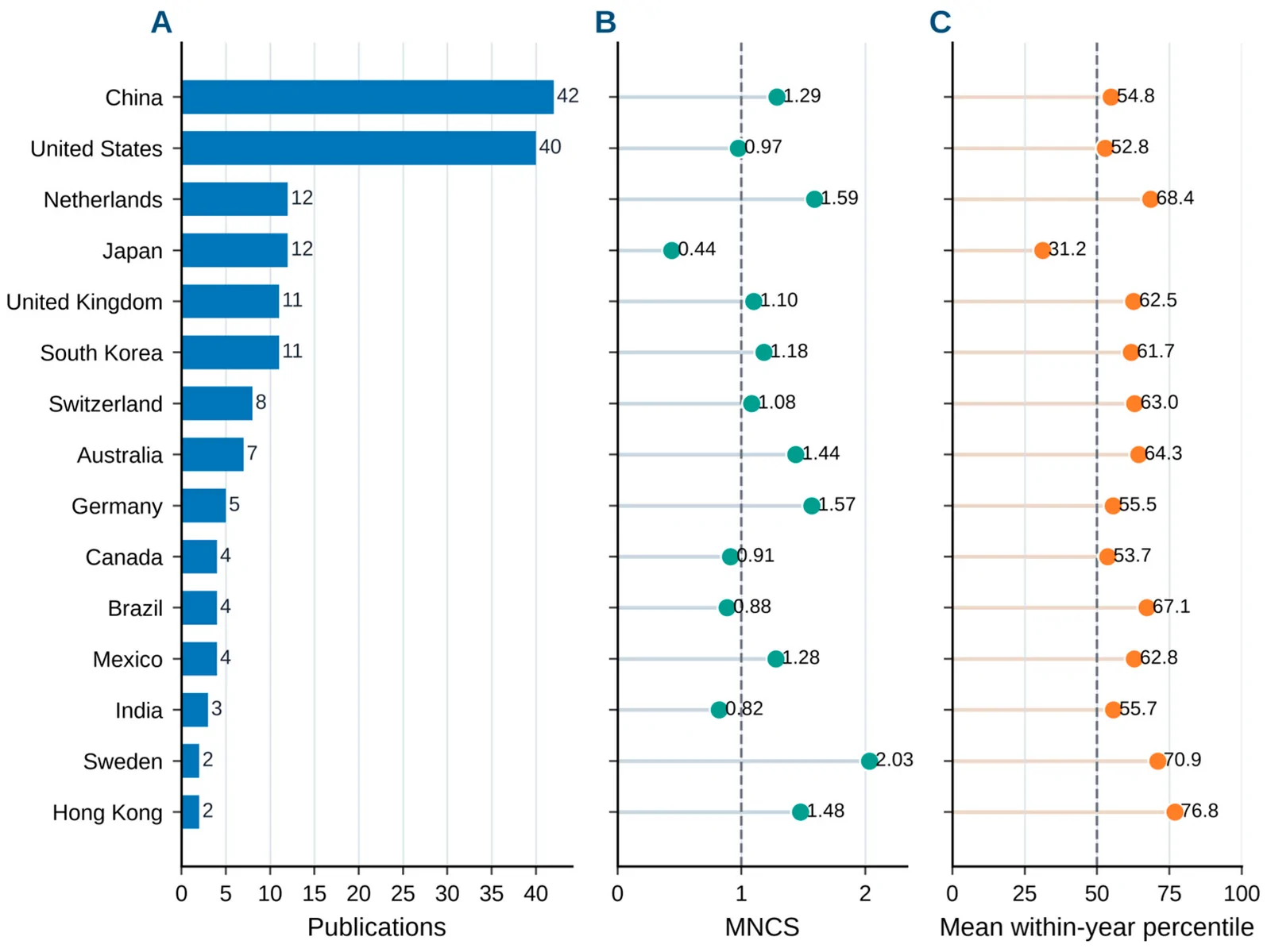
Country/region publication output and within-corpus citation profile. Countries are ranked by whole-counted publications (**A**); accompanying points show MNCS (**B**) and mean within-year percentile (**C**). The indicators are derived from the released record-country membership table and represent descriptive, internally age-adjusted citation patterns rather than field-normalized impact.

### 3.4. Institutional Productivity

Institution mappings were available for all 140 records with nonblank affiliation strings and resolved to 386 explicit organization-level labels. The Chinese Academy of Medical Sciences and Peking Union Medical College ranked first with 13 whole-counted publications. Shanghai Ninth People’s Hospital, Shanghai Jiao Tong University School of Medicine, Plastic Surgery Hospital, and University Medical Center Utrecht each contributed eight, and ETH Zurich contributed six (Table 2; Figure 4). Affiliated entities are retained at the organizational level stated in the source affiliation and should not be summed without record-level deduplication.

**Figure 4:**
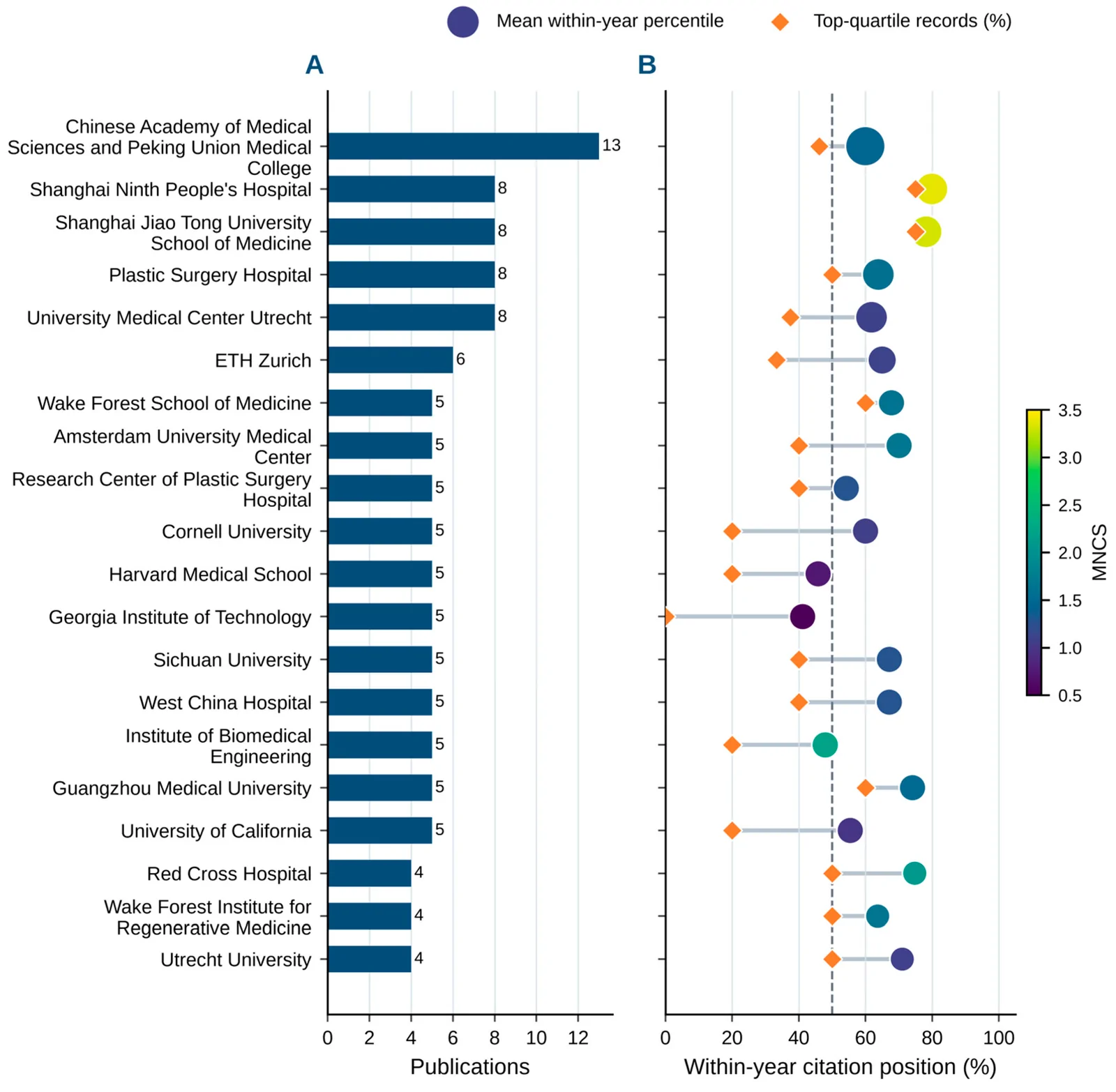
Institutional output and internally age-adjusted citation indicators. (**A**) shows whole-counted publication totals for the top 20 institution labels. (**B**) connects each institution’s mean within-year percentile (circles) with its percentage of top-quartile records (diamonds); circle area represents publication count and circle color represents MNCS.

Normalized metrics varied across the leading institutions. Among high-output institutions, MNCS ranged from 1.11 at University Medical Center Utrecht to 3.38 at Shanghai Ninth People’s Hospital; the latter estimate was based on eight records, six of which were at or above the 75th percentile within their publication years. Small groups and overlapping affiliations can materially affect these estimates; the results are therefore descriptive rather than evaluative.

### 3.5. Authors

The corpus contained 959 distinct submitted author labels, of which 162 appeared in at least two publications. Haiyue Jiang had the largest output with eight publications, followed by Yilin Cao with seven. Guangdong Zhou, Yu Liu, Jos Malda, Jason A. Spector, Xia Liu, Lawrence J. Bonassar, and Zhengyong Li each appeared on six publications (Table 3; Figure 5). Author labels were counted once per record; residual ambiguity cannot be excluded in the absence of complete persistent author identifiers.

**Figure 5:**
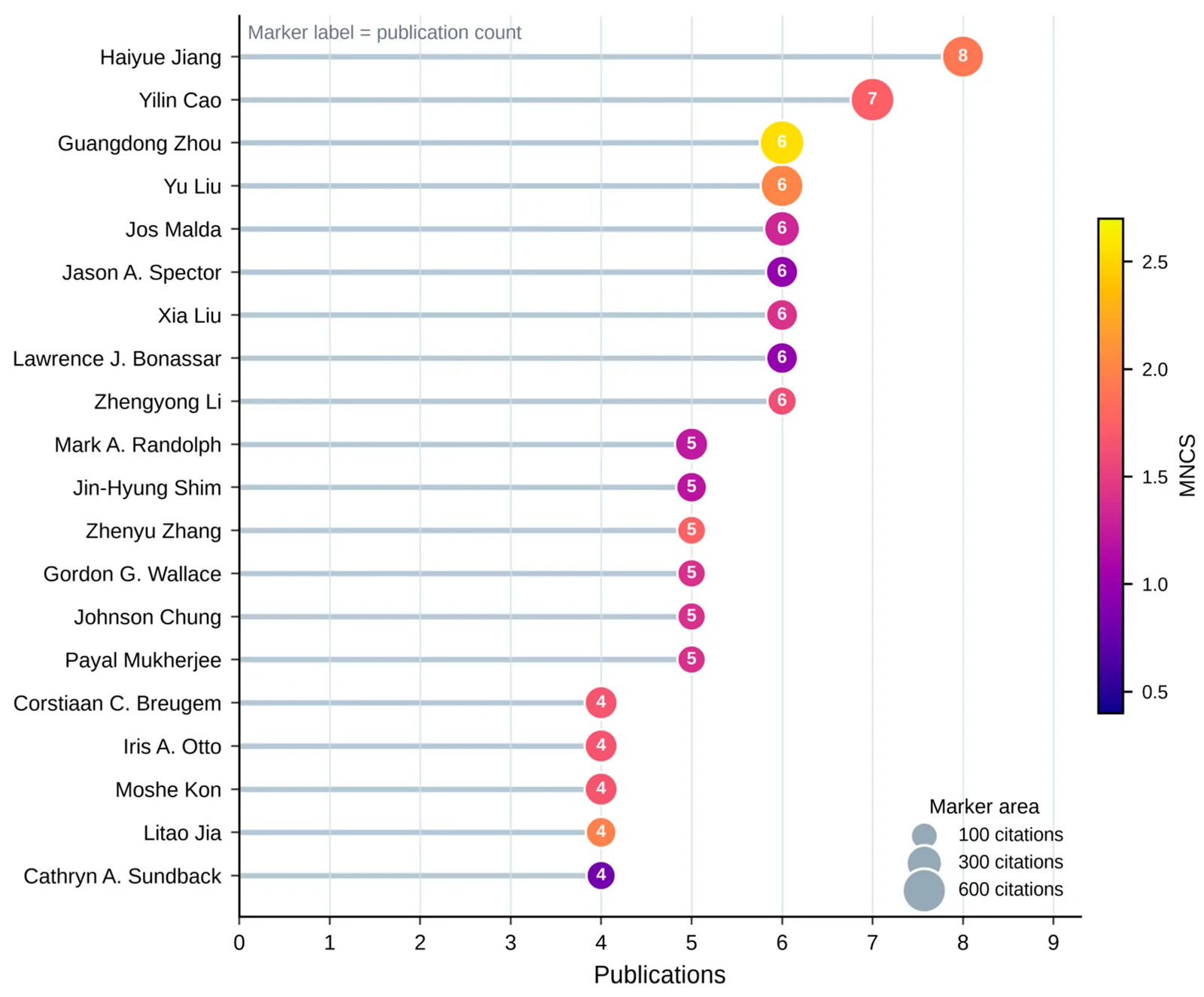
Output and internally age-adjusted citation patterns for the 20 most productive author labels. Each author is positioned by publication count; marker size represents total citations, and marker color represents MNCS. The plot is a descriptive record-membership summary and is subject to author-name ambiguity and whole-counting limitations.

Among the most productive authors, MNCS was 2.54 for Guangdong Zhou, 2.00 for Yu Liu, 1.92 for Haiyue Jiang, and 1.75 for Yilin Cao. These values reflect the publication-year composition and citation distribution of each author’s records within this corpus and should not be interpreted as individual research quality or intellectual leadership.

### 3.6. Journal Distribution

The 169 publications appeared in 103 standardized journals, and no journal contributed more than five records. *Journal of Biomedical Materials Research Part B: Applied Biomaterials*, *International Journal of Molecular Sciences*, *PLoS ONE*, and *The Laryngoscope* each published five records. *Biofabrication*, *Materials Today Bio*, *Journal of Tissue Engineering and Regenerative Medicine*, *ACS Omega*, *Plastic & Reconstructive Surgery*, and *Journal of Craniofacial Surgery* each published four (Figure 6). This dispersion reflects the intersection of reconstructive surgery, biomaterials, cartilage biology, and biofabrication.

**Figure 6:**
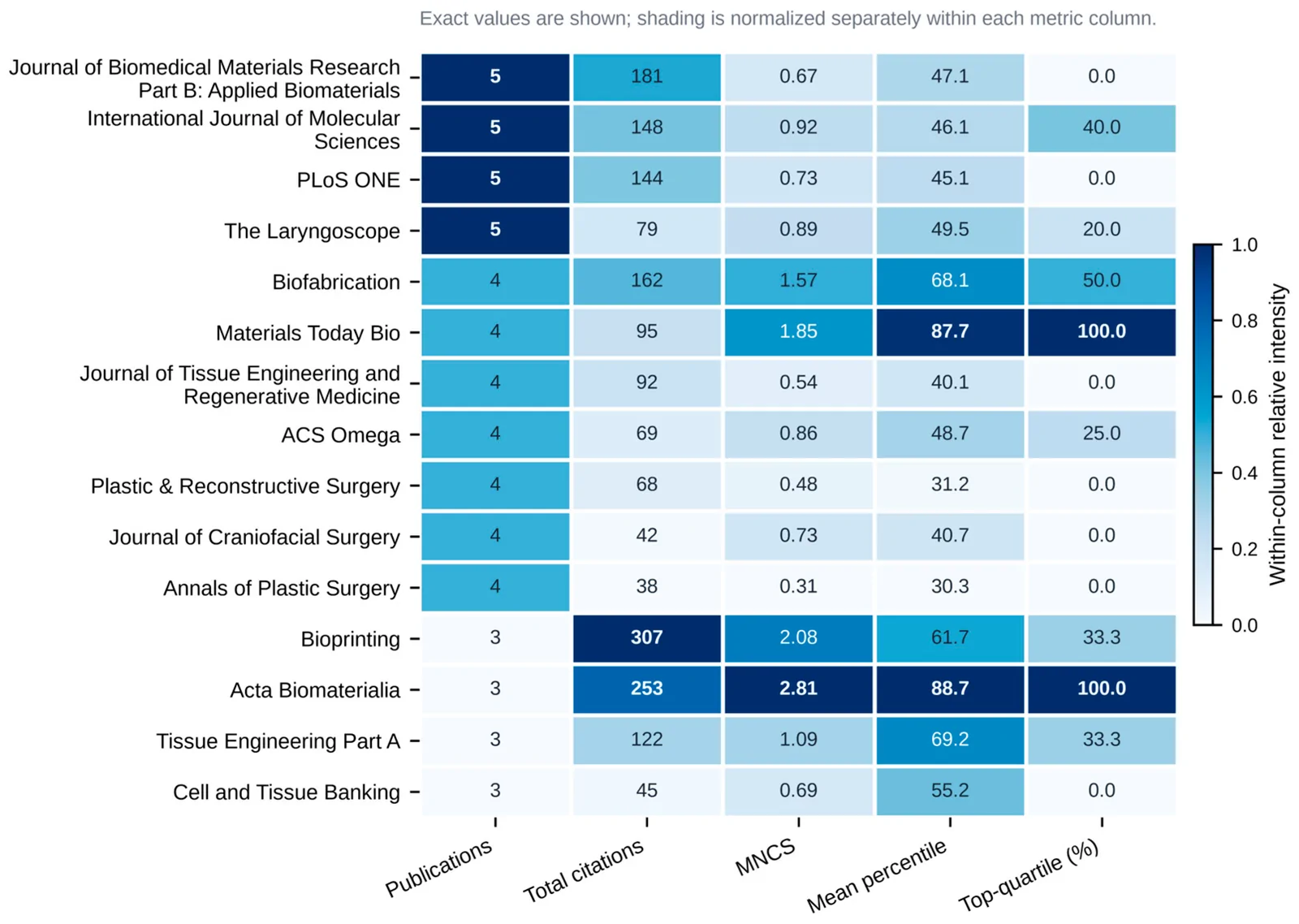
Journal output and within-corpus citation indicators. The 15 journals with the largest publication totals are shown as a heatmap-style profile across publications, total citations, MNCS, mean within-year percentile, and top-quartile proportion. Values are descriptive and should be interpreted in view of the small number of records per journal.

Among the journals with at least four records, MNCS was 1.85 for *Materials Today Bio* and 1.57 for *Biofabrication*, compared with 0.67 for *Journal of Biomedical Materials Research Part B* and 0.48 for *Plastic & Reconstructive Surgery*. Each estimate was based on only four or five papers and may reflect topic and cohort composition; it should not be interpreted as a venue effect or publication recommendation.

### 3.7. Highly Cited Studies

The ten records with the highest raw citation counts received 1526 citations, accounting for 35.6% of all corpus citations at the snapshot (Table 4) [16,17,18,19,20,21,22,23,24,25]. Zhou et al. (2018) [16] ranked first with 319 citations (Y-NCS 5.18; within-year percentile 96.9), followed by Martínez Ávila et al. (2016) [17] with 274 citations (Y-NCS 4.87; percentile 95.8) and Martínez Ávila et al. (2014) [19] with 163 citations (Y-NCS 3.51; percentile 95.0). All ten records also ranked highly within their publication-year cohorts.

The highly cited set included studies of patient-specific auricular cartilage regeneration, chondrocyte-laden nanocellulose hydrogels, cartilage-derived extracellular-matrix bioinks, decellularized scaffolds, and biofabrication-based reconstruction. It spanned publications from 2014 to 2022, indicating that both citation window and unusually high within-cohort citation performance contributed to the raw ranking. The complete top-50 table provides Record_ID, citation snapshot, Y-NCS, percentile, and DOI.

### 3.8. Conceptual Structure of the Field

Author keywords were available for 91 of 169 publications (53.8%). After HTML decoding, controlled normalization, and within-record deduplication, the dataset contained 470 record-term occurrences. Fifty normalized terms appeared in at least two records and accounted for 255 occurrences. The most frequent terms were tissue engineering (28 records), 3D printing (23), microtia (22), auricular reconstruction (19), scaffold (11), cartilage (9), auricular cartilage (8), and extracellular matrix (8).

Applying the prespecified co-occurrence threshold generated a 50-term matrix. When displayed edges were restricted to term pairs co-occurring in at least two records, 29 terms remained non-isolated and formed 58 weighted edges (Figure 7). Louvain partitioning identified five communities. Their highest-frequency anchors were microtia, polycaprolactone, and cartilage tissue engineering in Community 1; 3D printing and auricular reconstruction in Community 2; tissue engineering and cartilage in Community 3; extracellular matrix and bioink in Community 4; and scaffold, collagen, and decellularization in Community 5. These labels summarize an algorithmic partition rather than predetermined thematic categories.

**Figure 7:**
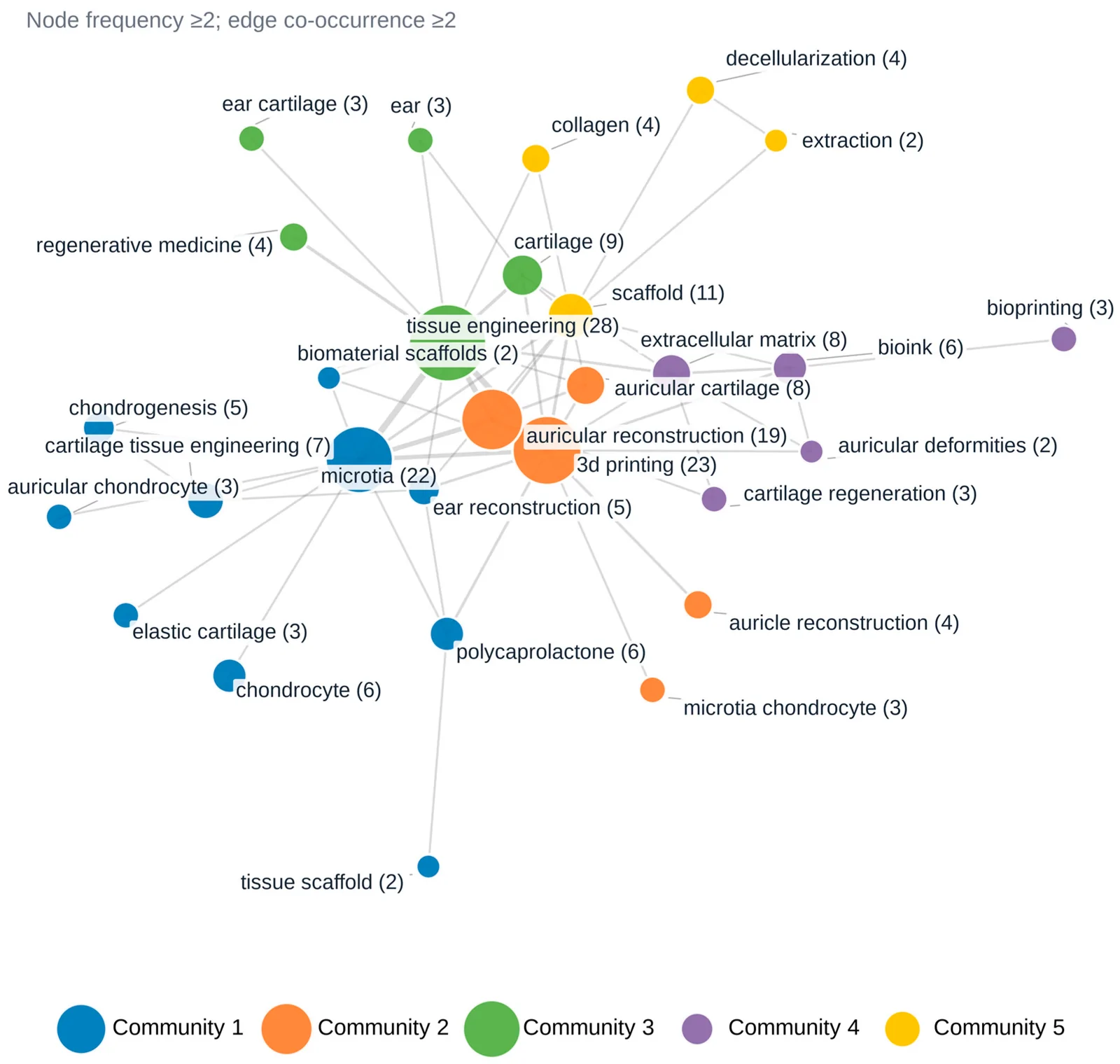
Reproducible author-keyword co-occurrence network. Terms occurring in at least two records entered the complete 50-term matrix; the displayed graph retains weighted edges with co-occurrence of at least two and contains 29 non-isolated nodes and 58 edges. Node area is proportional to record frequency, edge width to co-occurrence weight, and color to NetworkX 3.6.1 Louvain community (resolution 1.0; seed 42). Weighted spring-layout coordinates used seed 42 and 500 iterations.

### 3.9. Temporal Evolution of Author Keywords

Frequently used terms spanned most of the observation period. Tissue engineering appeared from 2014 to 2025 in 28 records, microtia from 2014 to 2025 in 22, and 3D printing from 2017 to 2025 in 23 (Figure 8). Bioink first appeared in the retained author-keyword set in 2020 and occurred in six records; decellularization also first appeared in 2020 and occurred in four.

**Figure 8:**
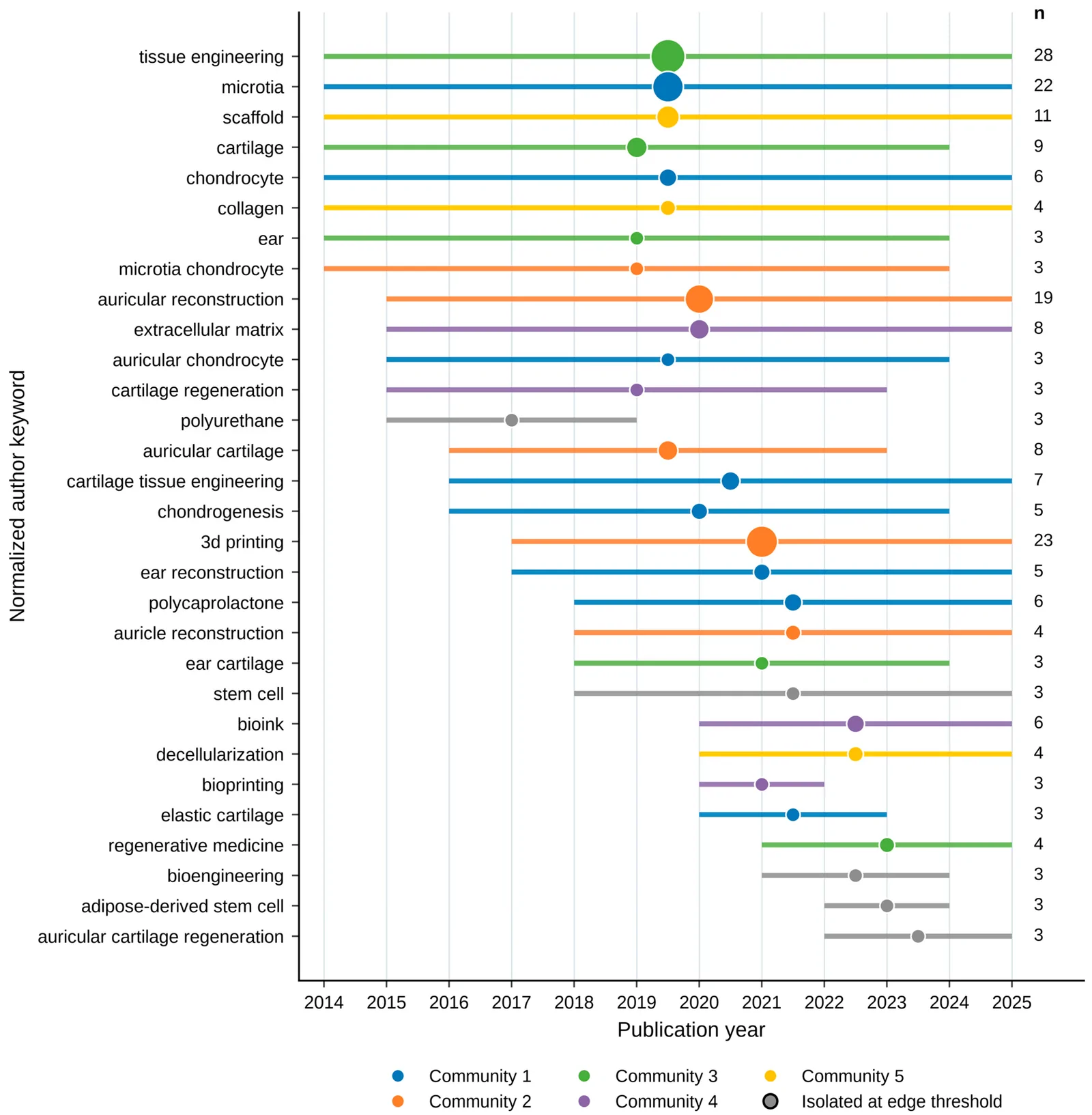
Temporal span of the 30 most frequent normalized author keywords. Lines extend from the first to the last occurrence year, marker area is proportional to record frequency, color follows Figure 7 community where available, and the right-hand value gives record frequency. Terms isolated by the Figure 7 edge threshold can appear because Figure 8 is based on frequency rather than connectivity.

The descriptive trend index identified small differences in term shares between periods (Figure 9). Bioink had *n*1 = 0 and *n*2 = 6, corresponding to 6/336 − 0/134 = +0.0179. Decellularization and regenerative medicine each had *n*1 = 0 and *n*2 = 4, yielding +0.0119. In the negative direction, polyurethane had an index of −0.0224 and tissue engineering −0.0211. Because the index is a share difference and author keywords were available for only 91 records, the results indicate modest changes in author terminology rather than a statistically established field-wide transition.

**Figure 9:**
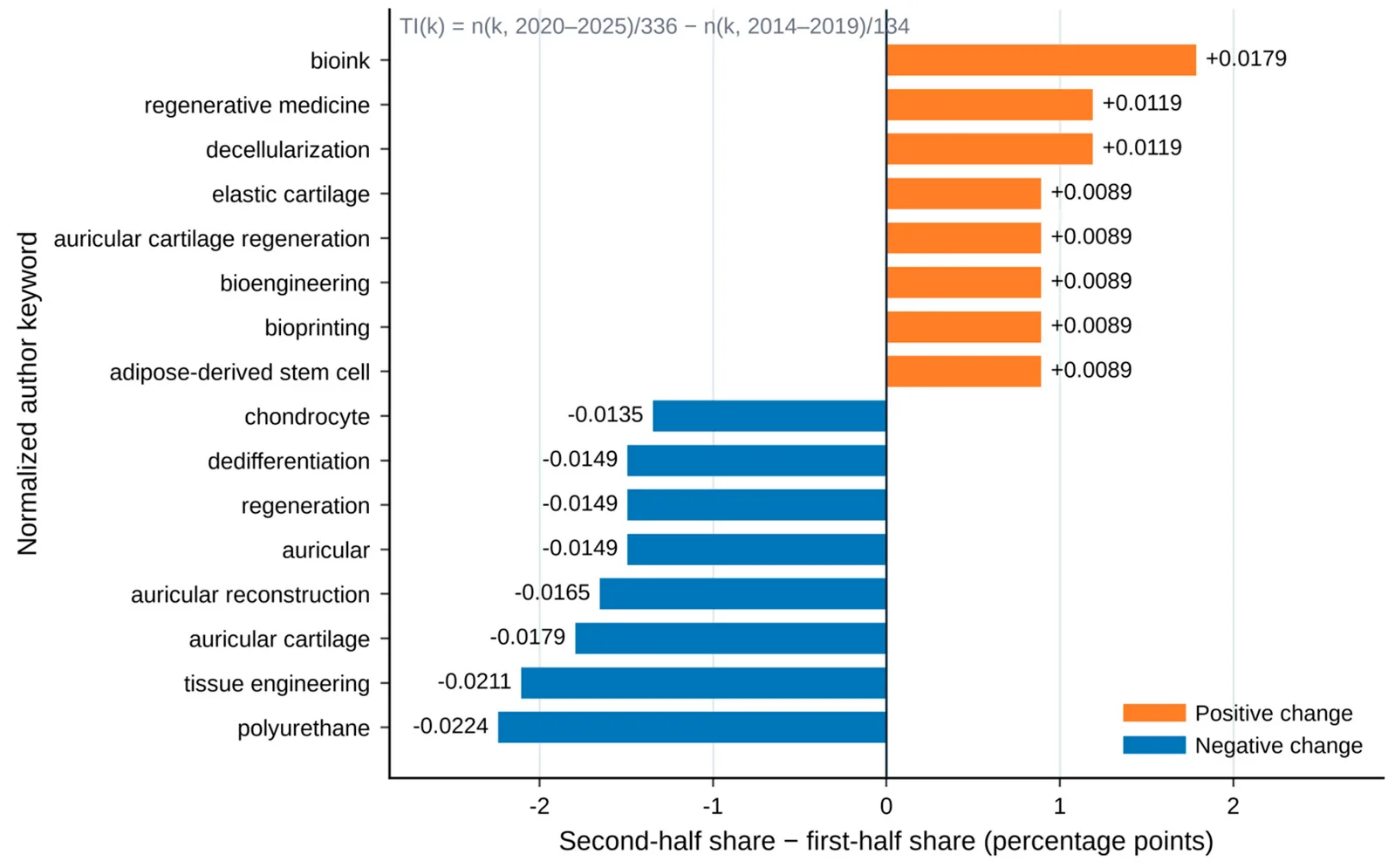
Largest positive and negative descriptive author-keyword share differences. For each of the 50 terms occurring in at least two records, TI(k) = *n*(k, 2020–2025)/336 − *n*(k, 2014–2019)/134. Bars show percentage-point differences and labels show the exact index. The index is not a significance test or burst statistic.

### 3.10. Citation-Age Sensitivity

Annual output increased from 10 publications in 2014 to 20 in 2025, but the trajectory was non-monotonic (Figure 10). The whole-corpus median CPY was 2.50 citations per publication-year (interquartile range, 1.33–5.33). Annual median CPY ranged from 1.00 in 2019 and 2025 to 4.81 in 2018; wide annual interquartile ranges indicated substantial within-cohort heterogeneity. The recent decline in median CPY occurred despite high publication output and is consistent with shorter and only approximately measured citation windows.

Raw citation concentration remained marked: the ten most cited records received 1526 citations, or 35.6% of the corpus total. However, CPY, Y-NCS, and within-year percentile did not provide identical rankings, confirming that fixed-snapshot citations reflect both exposure time and record-specific citation accumulation. Accordingly, the original fixed-snapshot highly cited record analysis remains in Table 4 as a descriptive measure of citation concentration, and no citation measure was used to infer scientific or clinical superiority.

**Figure 10:**
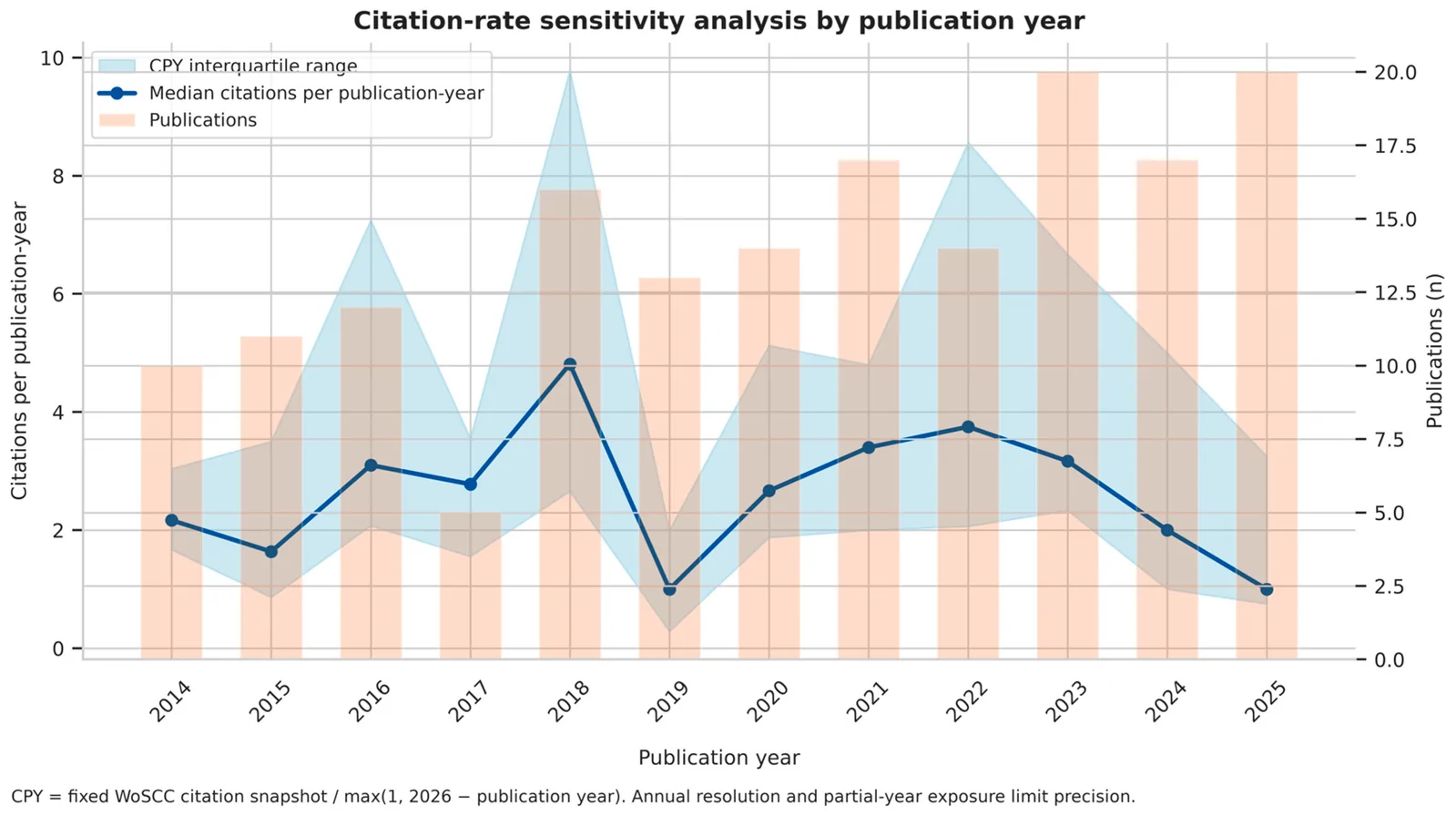
Publication output and citation-rate sensitivity analysis by publication year. Bars show the number of included records by Web of Science publication year. The line shows the median citations per publication-year (CPY), and the shaded band shows the interquartile range. CPY was calculated as the fixed 12 June 2026 WoSCC citation count divided by max(1, 2026 − publication year) because only annual publication dates were consistently available. CPY is reported alongside Y-NCS and within-year citation percentiles as a descriptive age-sensitivity measure and is not interpreted as research quality or clinical value.

### 3.11. Evidence Maturity and Technical Routes

Evidence maturity was predominantly preclinical (Figure 11A; Table 5). Among 143 Articles, 40 (28.0%) were in vitro only and 41 (28.7%) reached small-animal testing. Eight (5.6%) were ex vivo only, four (2.8%) reached large-animal testing, and nine (6.3%) reported human clinical evidence. Three (2.1%) were methods/modeling studies. The available title/abstract evidence was insufficient to assign a stage to 38 Articles (26.6%); these records were retained rather than forced into a maturity category.

**Figure 11:**
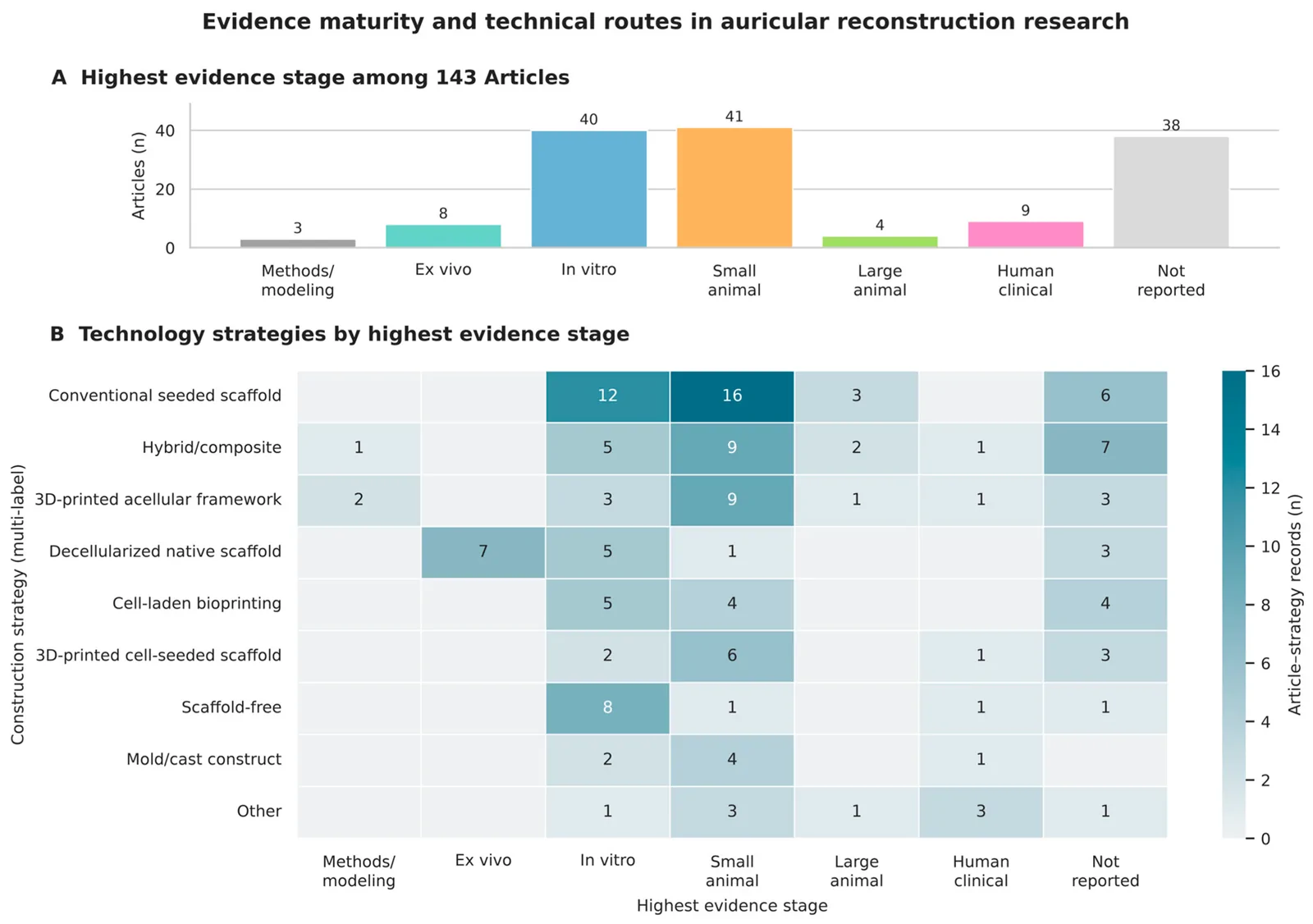
Evidence maturity and technical routes. (**A**) Highest evidence stage of the 143 Articles. (**B**) Multi-label construction strategies by stage. “Not reported” denotes insufficient record-level information for a defensible assignment.

Only 13 Articles (9.1%) reached either large-animal or human evidence, representing 12.4% of the 105 stage-classifiable Articles. Of the nine human studies, eight were first-in-human or case-series level and only one was comparative clinical research. Thus, the literature contained several important translational examples, including patient-specific ear-shaped cartilage and printed implant studies, but the overall evidence base did not support broad claims of clinical readiness.

The field did not converge on a single technical platform (Figure 11B). Conventional seeded scaffolds were the most frequent strategy (37 Articles, 25.9%), followed by hybrid or composite systems (25, 17.5%), 3D-printed acellular frameworks (19, 13.3%), decellularized native scaffolds (16, 11.2%), cell-laden bioprinting (13, 9.1%), 3D-printed cell-seeded scaffolds (12, 8.4%), and scaffold-free strategies (11, 7.7%). Only a small fraction of any strategy reached large-animal or human evaluation, and no strategy dominated the advanced-evidence strata.

Natural hydrogels or polymers and synthetic polymers were each identified in 57 Articles (39.9%); decellularized extracellular matrix or tissue appeared in 25 (17.5%) and reinforced composites in 23 (16.1%). Auricular or microtia chondrocytes were the most frequent cell strategy (48 Articles, 33.6%). Cell-free or acellular approaches and non-auricular chondrocytes each occurred in 25 Articles (17.5%); mixed-cell systems and mesenchymal/bone-marrow stromal cells each appeared in 14 (9.8%), adipose-derived stromal cells in 13 (9.1%), and other stem/progenitor cells in 11 (7.7%).

Construct scale revealed a further translational bottleneck. Fifty-five Articles (38.5%) studied small specimens, whereas 32 (22.4%) evaluated ear-shaped constructs, 11 (7.7%) evaluated full-scale auricles, and nine (6.3%) reported clinical reconstruction. Consequently, anatomical resemblance at small or ear-shaped scale was substantially more common than evidence from full-size constructs or patients.

### 3.12. Scientific Endpoints and Reporting Completeness

Scientific outcome coverage was uneven (Figure 12A). Chondrogenesis or extracellular-matrix outcomes were explicitly reported in 84 Articles (58.7%), mechanical properties in 48 (33.6%), and cell viability or proliferation in 46 (32.2%). Shape fidelity or geometry was reported in 30 Articles (21.0%), inflammation or biocompatibility in 26 (18.2%), elastic-cartilage or elastin outcomes in 21 (14.7%), and post-implantation shape retention or contraction in 19 (13.3%).

**Figure 12:**
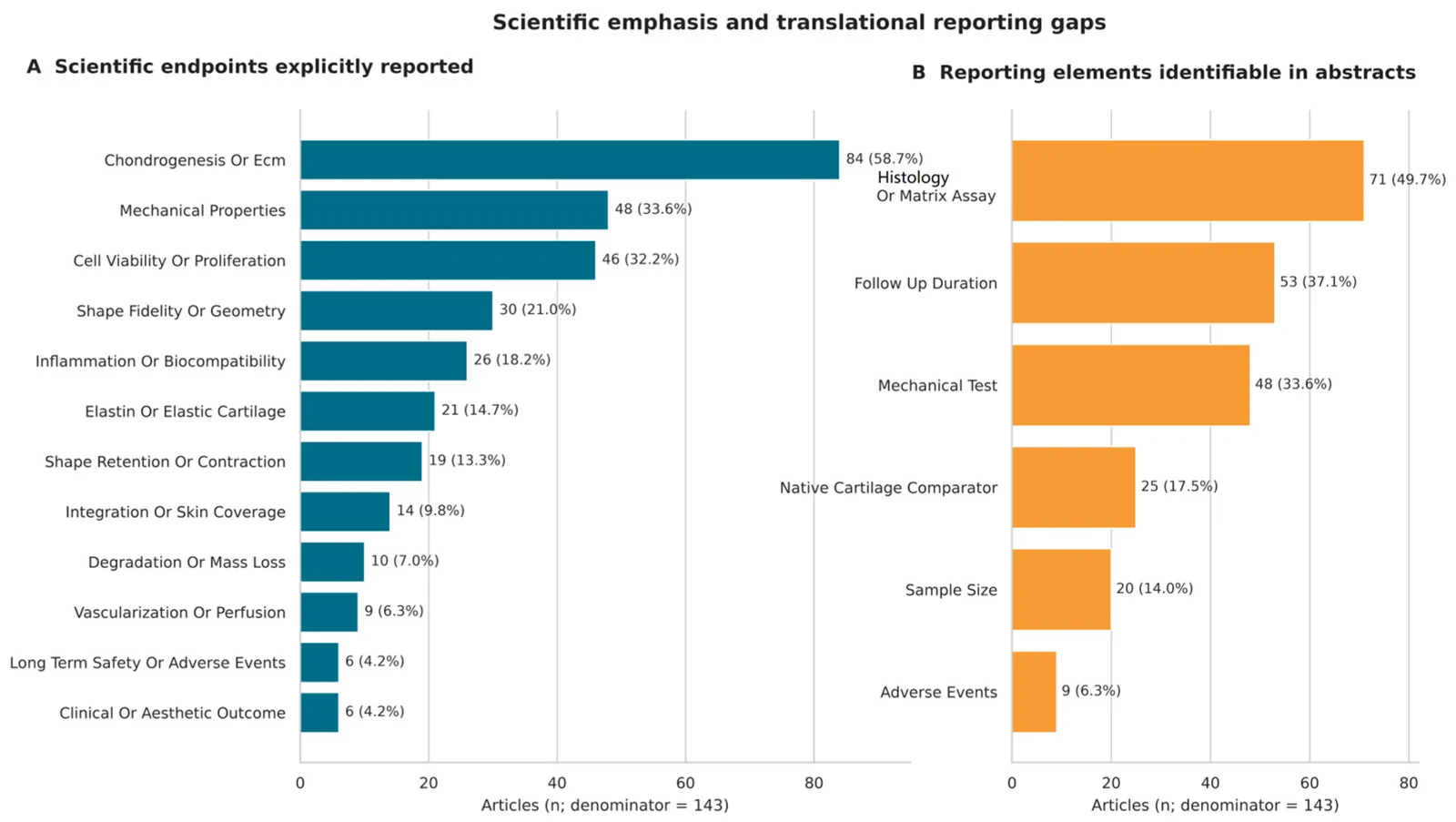
Scientific emphasis and translational reporting gaps. (**A**) Scientific endpoints explicitly reported in the titles or abstracts of the 143 Articles. (**B**) Reporting elements that could be identified from the abstracts. Counts are conservative and describe abstract-level visibility rather than full-text methodological quality; an uncounted item may have been assessed but not reported in the abstract.

Endpoints closest to long-term clinical performance were less visible. Host integration or skin coverage appeared in 14 Articles (9.8%), degradation in 10 (7.0%), vascularization or perfusion in nine (6.3%), clinical or aesthetic outcomes in six (4.2%), and long-term safety or adverse events in six (4.2%). The literature therefore emphasized cartilage formation and early construct performance more often than durable integration, vascular support, patient-facing outcomes, or safety.

Abstract-level reporting also limited cross-study comparison (Figure 12B). Histology or matrix assays were identifiable in 71 Articles (49.7%), follow-up duration in 53 (37.1%), and mechanical testing in 48 (33.6%). A native-cartilage comparator was identifiable in 25 (17.5%), sample size in 20 (14.0%), and adverse-event reporting in nine (6.3%). These values do not establish that uncounted studies omitted the methods; they show that clinically important information was often not visible in the abstract-level record used for mapping.

### 3.13. Translational Barriers and Advanced-Evidence Records

Explicit barrier coding supported a field-specific rather than purely bibliometric interpretation (Figure 13A). Cell supply, expansion, or dedifferentiation was identified in 22 of 169 records (13.0%); mechanical instability in 16 (9.5%); and shape loss or shrinkage in 14 (8.3%). Inflammation or immunogenicity appeared in 11 records (6.5%), insufficient long-term evidence in eight (4.7%), and regulatory or clinical-workflow barriers in seven (4.1%). Vascularization or nutritional support was explicit in six records (3.6%); degradation mismatch and skin or soft-tissue coverage each appeared in five (3.0%); and manufacturing reproducibility in three (1.8%). Because barriers were counted only when explicit, these values are conservative and should not be interpreted as prevalence estimates.

**Figure 13:**
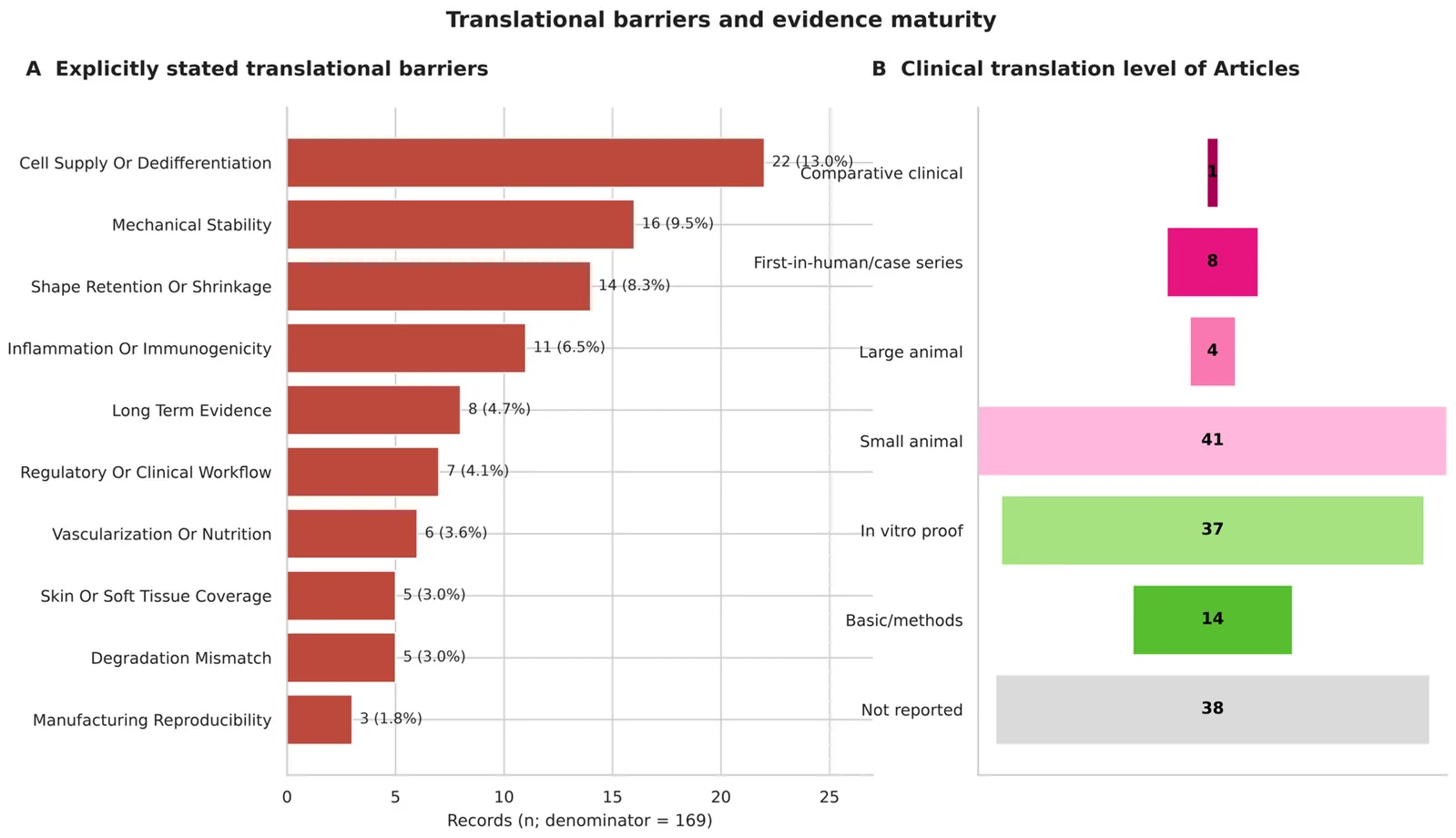
Translational barriers and evidence maturity. (**A**) Barriers explicitly described as limitations, challenges, unresolved problems, or design targets in the titles or abstracts of the 169 records. (**B**) Highest clinical translation level assigned to the 143 Articles. Reviews were not scored. “Not reported” denotes insufficient record-level information for assignment.

The 13 large-animal or human records illustrate both progress and fragmentation (Table 6; Figure 13B). Human evidence included autologous cell-engineered cartilage, printed implants, porous polyethylene frameworks, and cartilage-scaffold procedures, but sample sizes, comparators, and follow-up varied. Large-animal evidence was limited to four records and did not establish a single preferred material–cell–fabrication combination. High citation rates occurred in some translational records, particularly the first clinical application of patient-specific ear-shaped cartilage, but citation performance and evidence maturity were not interchangeable.

The complete record-level table for all 13 advanced-evidence Articles, including title, year, model, construct scale, follow-up, CPY, Y-NCS, within-year percentile, evidence quotation, and confidence, is provided in the Advanced_Evidence worksheet of Supplementary Information S2.

## 4. Discussion

The bibliometric and record-level analyses provide complementary perspectives on the development of auricular bioengineering. Research activity increased and diversified across reconstructive surgery, biomaterials, cartilage engineering, and biofabrication, with recent terminology increasingly emphasizing bioinks, decellularized matrices, and patient-specific manufacturing. However, most experimental evidence remained confined to in vitro or small-animal studies, and relatively few investigations evaluated full-scale constructs, long-term shape retention, tissue integration, or clinical outcomes. The findings therefore indicate that the main challenge is no longer the generation of ear-shaped prototypes, but the reproducible conversion of biological and manufacturing advances into mechanically stable, anatomically faithful, well-integrated constructs that can withstand clinically relevant implantation and follow-up.

### 4.1. Bibliometric Landscape and Evolution of the Field

The increase from 10 publications in 2014 to 20 in 2025, together with the concentration of more than half of the corpus in 2021–2025, indicates sustained interest in engineered alternatives for auricular reconstruction. The annual trajectory was nevertheless non-monotonic, suggesting gradual consolidation rather than uninterrupted exponential expansion. China and the United States contributed the largest whole-counted outputs, while institutions and authors from several regions formed a distributed collaboration landscape. These patterns are consistent with broader investment in additive manufacturing and regenerative medicine, but they should not be interpreted as evidence of national or institutional superiority. Publication counts reflect research capacity, funding, database coverage, and collaboration structure, whereas the dispersed journal distribution reflects the interdisciplinary location of the field between reconstructive surgery, biomaterials science, cartilage biology, and biofabrication [12].

The keyword network further illustrates this interdisciplinary structure. Its five communities connected microtia and auricular reconstruction with fabrication technologies, scaffold design, cartilage biology, extracellular-matrix approaches, and cell-containing constructs. The increasing visibility of bioink and decellularization after 2020 is technically consistent with efforts to combine printable hydrogels with tissue-specific biochemical cues [26,27,28,29]. At the same time, the prominence of reinforcement, polymers, and composite systems reflects the need to compensate for the limited mechanical stability of many cell-compatible matrices [30,31]. These shifts do not demonstrate replacement of one platform by another; rather, they show that the field is attempting to integrate biological specificity, printability, and structural support within the same construct.

These temporal patterns require an additional metadata qualification. Usable author keywords were available for 91 of the 169 included records, and their availability can vary with journal indexing practices and article format. The keyword-bearing subset may therefore over-represent terminology used in biomaterials and biofabrication articles relative to clinical surgical reports. Because the missing author-keyword field cannot be reconstructed reliably, neither the direction nor the magnitude of this sampling effect can be estimated. The trend indices should consequently be read as descriptive changes within the keyword-supplying subset, not as field-wide estimates of methodological adoption or evidence that one platform has displaced another.

Citation patterns provide a complementary but restricted view of influence. Frequently cited studies introduced patient-specific cartilage constructs, nanocellulose systems, decellularized scaffolds, and extracellular-matrix bioinks that helped define major technical directions [16,17,18,19,20,21,22,23,24,25]. Their visibility reflects scientific relevance, but also publication age, journal exposure, and the size of the citing community. The non-monotonic annual pattern of citations per publication-year and the changes produced by within-year normalization confirm that raw citation totals favor earlier records. Citation and entity metrics are therefore most useful for locating influential work and research communities; they cannot determine experimental rigor, platform efficacy, or clinical readiness.

### 4.2. Engineering Strategies and the Absence of a Dominant Platform

The distribution of technical routes shows that auricular reconstruction has not converged on a single material–cell–fabrication strategy. Conventional seeded scaffolds were most frequent, but hybrid composites, printed acellular frameworks, decellularized scaffolds, cell-laden bioprinting, printed cell-seeded constructs, and scaffold-free approaches were all represented. This diversity reflects a persistent engineering trade-off. Natural hydrogels and cartilage-derived extracellular matrices can provide hydrated, cell-compatible environments and tissue-specific biochemical signals, but often lack the immediate stiffness and long-term dimensional stability required for a thin, highly contoured auricle [22,26,27,28,29]. Synthetic frameworks can improve handling, pore control, and shape preservation, yet excessive stiffness, poorly matched degradation, chronic inflammation, and weak tissue interfaces may compromise remodeling. Reinforced and interpenetrating systems attempt to combine these advantages, although increased architectural complexity also complicates manufacturing, sterilization, quality control, and degradation–regeneration matching [30,31,32].

Nanocellulose is one candidate reinforcing phase because it can alter rheology and improve shape fidelity or mechanical stability in otherwise weak hydrated matrices. In a gastric tissue-derived dECM bioink, cellulose nanoparticles were used to increase matrix stiffness and improve biophysical characteristics of printed constructs [33]. In cartilage-oriented bioinks, nanocellulose has also been associated with increased viscosity, shear recovery, printability, and mechanical strength, although concentration, crosslinking, and cell response remain formulation-specific [34]. Decellularized cellulose-rich seaweed scaffolds likewise illustrate that decellularization can preserve architecture while substantially changing tensile behavior [35]. These non-auricular systems support the mechanistic rationale for examining nanocellulose–dECM composites, but they do not establish auricular safety, phenotype preservation, long-term degradation, or clinical benefit. Such composites should therefore be evaluated against native-cartilage mechanics and long-term shape retention rather than considered an established solution.

Cell selection introduces a second level of uncertainty. Auricular or microtia chondrocytes are attractive because they can produce tissue-relevant matrices, but the limited amount of donor cartilage and phenotype loss during expansion constrain their use. Co-culture with mesenchymal or adipose-derived stromal cells may reduce the required chondrocyte burden and support cartilage formation, as demonstrated in patient-derived ear-shaped constructs [16,23]. Conversely, acellular and decellularized approaches avoid ex vivo cell manufacturing but depend more heavily on host–cell recruitment, infiltration, matrix remodeling, and vascular support at the construct boundary. These alternatives solve different parts of the translational problem, and the present evidence does not identify a cell source that consistently performs across scale, mechanics, long-term stability, and clinical feasibility.

The lack of convergence should not be interpreted as failure of the field. It indicates that platform performance has usually been optimized along one or two dimensions rather than across the full set required for reconstruction. A biologically active hydrogel may form cartilage but lose contour; a reinforced framework may preserve geometry but remodel incompletely; a decellularized matrix may provide instructive cues but vary between batches; and a patient-specific construct may be manufacturable without yet being practical to sterilize, store, transport, or implant. Progress will therefore depend on comparative evaluation of integrated performance—including cell-source feasibility, elastic-cartilage phenotype, reproducible manufacture, regional mechanics, degradation, shape retention, host response, and surgical workflow—rather than on the publication frequency of individual materials or printing methods [6,32].

### 4.3. Scale, Mechanics, and Tissue Integration as Translational Bottlenecks

Construct scale is a critical determinant of whether experimental success is likely to translate. More than one-third of the Articles investigated small specimens, whereas fewer than one in ten evaluated a full-scale auricle. Small samples are appropriate for screening viability, matrix deposition, and local material behavior, but they do not reproduce the diffusion distances, unsupported contours, bending loads, regional thickness, thin skin envelope, or surgical handling of a human ear. This distinction is reinforced by the limited visibility of post-implantation shape retention and native-cartilage comparison in the mapped literature. Patient-specific cartilage reconstruction has demonstrated that a construct can develop cartilage-like matrix and retain gross auricular morphology while still undergoing clinically important contraction [16]. Likewise, polymer reinforcement can improve stability, but its benefit depends on whether scaffold degradation and tissue replacement preserve rather than destabilize the intended geometry [31].

Mechanical performance must therefore be considered regionally and longitudinally. The helix, antihelix, concha, and lobular transition do not experience identical loading or require identical stiffness, and a single bulk compression value cannot capture elastic recoil or contour preservation. Relevant assessment should relate biochemical maturation to three-dimensional geometric deviation, regional thickness, bending or compression behavior, elastic recovery, degradation, and change in projection over time. Native auricular cartilage is an important comparator because tissue-engineered constructs must reproduce not only cartilage matrix, but also the compliant mechanical behavior that distinguishes the external ear from articular or costal cartilage. The relative scarcity of these comparisons helps explain why many promising constructs remain difficult to compare across studies.

Tissue integration adds another interdependent constraint. Although mature auricular cartilage is avascular, a large engineered construct depends on diffusion and on a viable surrounding bed. Inadequate soft-tissue perfusion, inflammation, unstable interfaces, infection, or framework exposure can compromise an otherwise chondrogenic implant. Perfusion-decellularized ear scaffolds, vascularization studies, and integrated perichondrium–cartilage bioprinting illustrate possible strategies for supporting the construct–host interface [8,20,36]. The objective is not indiscriminate vascular invasion of cartilage, but formation of a stable cartilage–perichondrium–soft-tissue unit in which adjacent vascular support sustains the thin covering tissues while the cartilage compartment maintains phenotype and contour. The low frequency of vascular/perfusion, host-integration, and skin-coverage endpoints indicates that this interface remains underexamined, even though recent reviews identify inflammation, resorption, exposure, and long-term morphology as linked determinants of bioimplant success [32].

### 4.4. Evidence Maturity and Priorities for Clinical Translation

The evidence-stage distribution places these engineering challenges in a translational context. Eighty-one of 143 Articles were limited to in vitro or small-animal evidence, whereas only 13 reached large-animal or human evaluation and only one provided comparative clinical evidence. The available human studies demonstrate feasibility across cell-engineered cartilage, printed implants, and scaffold-based reconstruction, but predominantly involve small uncontrolled cohorts with heterogeneous follow-up and outcome reporting. Early clinical success is important, yet it does not establish comparative effectiveness, long-term safety, or reproducibility. The small number of large-animal studies is also consequential because scale-dependent nutrient limitations, soft-tissue complications, inflammatory remodeling, and mechanical failure may not become apparent in small ectopic models.

The endpoint distribution indicates that translational maturity is constrained as much by what is measured as by the number of studies. Cartilage matrix and cell outcomes were common, whereas vascular support, long-term shape, adverse events, clinical appearance, function, and patient-reported outcomes were uncommon. General animal-study rigor should follow established guidance on design, sample size, bias reduction, outcome definition, statistical analysis, animal characteristics, procedures, and results [37]. Auricular reconstruction additionally requires tissue-specific reporting of construct dimensions, regional mechanics, elastin and cartilage matrix, degradation, spatial viability, integration with perichondrial or fascial tissue, skin coverage, infection, exposure, contour stability, and revision. These variables link experimental performance to the practical reasons why an auricular implant succeeds or fails.

A staged translational pathway follows from these findings. Candidate platforms should first be compared using common biological, mechanical, geometric, degradation, and host-response outcomes. Systems that meet predefined performance thresholds should then be tested as reproducible full-scale auricles, followed by anatomically relevant, immunocompetent large-animal studies that examine surgical handling, implantation-bed integration, soft-tissue coverage, inflammation, infection, and long-term contour. Clinical studies should report standardized aesthetic, functional, safety, patient-reported, revision, and reoperation outcomes and, where feasible, compare engineered constructs with established autologous or alloplastic reconstruction [1,3,16]. Citation rate cannot substitute for this progression: CPY and within-year measures reduce publication-age distortion, but translational maturity ultimately depends on reproducible performance across successive experimental and clinical stages.

### 4.5. Strengths and Limitations

This study integrates a reproducible bibliometric map with a source-linked evidence map focused on scientific and translational content. The locked corpus, explicit eligibility criteria, stable record identifiers, released decision logs, controlled entity and keyword mappings, complete co-occurrence matrix, fixed analytical parameters, figure-source tables, executable code, and checksums support independent audit. Record-level coding extended the analysis beyond publication counts by characterizing technical routes, materials, cell systems, construct scale, evidence stage, endpoints, reporting elements, and explicitly stated barriers. Source quotations, confidence labels, a second coding pass for higher-risk records, and deterministic validation reduced internal inconsistency, while separate reporting of raw citations, CPY, year-normalized scores, and within-year percentiles made the limits of citation-based interpretation explicit.

Several limitations remain. The corpus was restricted to English-language Articles and Reviews indexed in WoSCC, and the original 1253-record screening-pool export was not retained; complete record-level traceability begins with the 294-record late-stage set. Cited-reference fields, Keywords Plus, and external subject-category baselines were unavailable, precluding co-citation, bibliographic-coupling, and external field-normalized analyses. Author keywords and affiliation strings were incomplete, entity disambiguation may retain residual error, and whole counting assigns full credit to every collaborating entity. The content map relied primarily on titles and abstracts, including 24 records with insufficient abstract information, and evidence stage could not be assigned for 38 Articles. Endpoint and reporting counts may therefore underestimate information available only in full text.

Finally, the evidence map was designed to characterize maturity and gaps rather than to estimate treatment effects. Heterogeneity in constructs, models, endpoints, and follow-up precluded meta-analysis, and the coding framework does not replace full-text risk-of-bias assessment. The findings support conclusions about the distribution of evidence, recurrent engineering barriers, and priorities for validation, but they do not establish the superiority, safety, or clinical effectiveness of any individual platform. Within these boundaries, the combined analysis provides a reproducible account of how the field is organized and why durable, full-scale, clinically validated auricular reconstruction remains an unresolved bioengineering objective.

## 5. Conclusions

From 2014 to 2025, 169 publications on 3D printing and tissue engineering for auricular reconstruction accumulated 4291 citations, with annual output rising from 10 to 20. China and the US led in output, with varied age-adjusted citation profiles. Keyword analysis revealed 50 recurrent terms and a five-cluster network; bioink and decellularization gained share post-2020, but clinical maturity is lacking. Progress demands standardized reporting, comparative evaluations, and evidence on vascularization, mechanics, shape retention, and safety.

The field remains preclinical: 81 of 143 Articles were in vitro/small-animal, and 13 reached large-animal/human, with only one comparative. Cartilage matrix and mechanics were over-studied vs. integration, full-scale shape, outcomes, and safety. Barriers: Cell supply, instability, shrinkage. Priorities: Reproducible full-scale constructs, native benchmarks, long-term stability, degradation–regeneration balance, implant-bed/skin integration, immunocompetent large-animal validation, adverse-event reporting, and clinical comparisons. Actions: Adopt benchmarks, longitudinal reporting, and comparative evaluation.

Bibliometrics aid in locating contributors and reducing citation-age bias, but not quality. The evidence map outlines achievements, gaps, and needed experiments for clinically durable auricular reconstruction.

## Figures and Tables

**Table 1:** Top 10 countries/regions by whole-counted publication total.

Mean Within-Year Percentile	MNCS	Percentage of Corpus (%)	Publications	Country/Region	Rank
54.8	1.29	24.9	42	China	1
52.8	0.97	23.7	40	United States	2
68.4	1.59	7.1	12	The Netherlands	3
31.2	0.44	7.1	12	Japan	4
62.5	1.10	6.5	11	United Kingdom	5
61.7	1.18	6.5	11	Republic of Korea	6
63.0	1.08	4.7	8	Switzerland	7
64.3	1.44	4.1	7	Australia	8
55.5	1.57	3.0	5	Germany	9
53.7	0.91	2.4	4	Canada	10

**Table 2:** Top 10 institution labels by whole-counted publication total.

Top-Quartile Records (%)	Mean Within-Year Percentile	MNCS	Publications	Institution	Rank
46.2	59.9	1.48	13	Chinese Academy of Medical Sciences and Peking Union Medical College	1
75.0	79.9	3.38	8	Shanghai Ninth People’s Hospital	2
75.0	78.1	3.31	8	Shanghai Jiao Tong University School of Medicine	3
50.0	63.8	1.58	8	Plastic Surgery Hospital	4
37.5	61.8	1.11	8	University Medical Center Utrecht	5
33.3	65.0	1.13	6	ETH Zurich	6
60.0	67.8	1.64	5	Wake Forest School of Medicine	7
40.0	70.1	1.68	5	Amsterdam University Medical Center	8
40.0	54.2	1.28	5	Research Center of Plastic Surgery Hospital	9
20.0	60.0	1.08	5	Cornell University	10

**Table 3:** Top 10 author labels by publication total.

Mean Within-Year Percentile	MNCS	Publications	Author	Rank
65.2	1.92	8	Haiyue Jiang	1
67.1	1.75	7	Yilin Cao	2
77.0	2.54	6	Guangdong Zhou	3
71.3	2.00	6	Yu Liu	4
70.6	1.32	6	Jos Malda	5
56.3	0.97	6	Jason A. Spector	6
60.3	1.40	6	Xia Liu	7
55.7	0.97	6	Lawrence J. Bonassar	8
74.4	1.62	6	Zhengyong Li	9
59.6	1.22	5	Mark A. Randolph	10

**Table 4:** Ten records with the highest raw WoSCC citation counts at the 12 June 2026 snapshot.

Within-Year Percentile	Y-NCS	Cited_By_Count	Journal	Year	Title	Rank
96.9	5.18	319	EBioMedicine	2018	In Vitro Regeneration of Patient-specific Ear-shaped Cartilage and Its First Clinical Application for Auricular Reconstruction	1
95.8	4.87	274	Bioprinting	2016	3D bioprinting of human chondrocyte-laden nanocellulose hydrogels for patient-specific auricular cartilage regeneration	2
95.0	3.51	163	Applied Microbiology and Biotechnology	2014	Biocompatibility evaluation of densified bacterial nanocellulose hydrogel as an implant material for auricular cartilage regeneration	3
96.4	4.71	143	Acta Biomaterialia	2020	A photo-crosslinkable cartilage-derived extracellular-matrix bioink for auricular cartilage tissue engineering	4
85.0	2.69	125	Biomaterials	2014	Regeneration of human-ear-shaped cartilage by co-culturing human microtia chondrocytes with BMSCs	5
90.6	1.77	109	Virtual and Physical Prototyping	2018	Aesthetic reconstruction of microtia: a review of current techniques and new 3D printing approaches	6
95.5	3.32	105	Biomedical Materials	2015	Preparation and characterization of a decellularized cartilage scaffold for ear cartilage reconstruction	7
96.4	4.25	101	Bioactive Materials	2022	Bioprinting and regeneration of auricular cartilage using a bioactive bioink based on microporous photocrosslinkable acellular cartilage matrix	8
84.4	1.56	96	Acta Biomaterialia	2018	Perfusion-decellularization of human ear grafts enables ECM-based scaffolds for auricular vascularized composite tissue engineering	9
86.4	2.88	91	Biofabrication	2015	Auricular reconstruction using biofabrication-based tissue engineering strategies	10

**Table 5:** Highest evidence stage of the 143 Articles.

Percentage of Articles (%)	n	Operational Interpretation	Evidence Stage
2.1	3	Fabrication, planning, or model development without biological evaluation identifiable in the record	Methods/modeling only
5.6	8	Testing using excised tissue or non-living biological material	Ex vivo only
28.0	40	Cell, matrix, scaffold, or construct evaluation without in vivo implantation	In vitro only
28.7	41	Highest identifiable stage involved a small-animal model	Small-animal in vivo
2.8	4	Highest identifiable stage involved a large-animal model	Large-animal in vivo
6.3	9	Human reconstruction, case series, or comparative clinical evidence	Human clinical
26.6	38	Available title/abstract evidence was insufficient for a defensible stage assignment	Not reliably assignable

**Table 6:** Summary of Articles reaching large-animal or human clinical evidence.

Main Limitation Visible in the Map	Evidence Represented	Percentage of Articles (%)	Articles, n	Evidence Group
Few studies; heterogeneous scale and follow-up; no comparative human outcome	Pig or other large-animal implantation	2.8	4	Large-animal in vivo
Predominantly small uncontrolled cohorts; variable follow-up and outcome reporting	Clinical reconstruction with engineered cartilage, printed implants, or alloplastic scaffolds	5.6	8	First-in-human or case series
Single study; short follow-up and no replicated comparative evidence	Human comparative reconstruction study	0.7	1	Comparative clinical

## Data Availability

The original contributions presented in this study are included in the article/Supplementary Materials; further inquiries can be directed to the corresponding author.

## Supplementary Materials

The following supporting information can be downloaded at: https://www.mdpi.com/article/10.3390/bioengineering13101095/s1.

## Abbreviations

3D	Three-Dimensional
BMSC	Bone Marrow Stromal Cell
CAGR	Compound Annual Growth Rate
CPY	Citations per Publication-Year
dECM	Decellularized Extracellular Matrix
ECM	Extracellular Matrix
MNCS	Mean Normalized Citation Score
NR	Not Reported/Not Reliably Assignable
TS	Topic Search
WoSCC	Web of Science Core Collection
Y-NCS	Year-Normalized Citation Score
