# Supplementary material for: Three-Dimensional Printing and Tissue Engineering in Auricular Reconstruction: Global Trends, Research Frontiers, and a Translational Evidence Map
Source: Bioengineering (Basel). 2026 Sep 22;13(10):1095. doi: 10.3390/bioengineering13101095 (PMC13701565; doi:10.3390/bioengineering13101095)
Supplement: Supplementary file 1 [file bioengineering-13-01095-s001.zip › Supplementary Information S1.pdf]

Supplementary Information 1

S1. Purpose and Analytical Scope

This document provides the full search, eligibility, data-processing, and reproducibility specifications supporting the manuscript. The analytical corpus contains 169 English-language, full-length journal publications indexed in the Web of Science Core Collection (WoSCC) from 2014 through 2025, comprising 143 Articles and 26 Reviews. All corpus-dependent statistics, tables, and figures derive from this locked dataset. Stable record identifiers, record-level eligibility decisions, entity memberships, keyword mappings, network matrices, trend calculations, figure-source tables, executable code, software versions, fixed parameters, and checksums are supplied with the submission.

S2. Search Strategy and Study Selection

The WoSCC topic search combined a fabrication and biofabrication concept block with an auricular anatomy and pathology block:

```
TS=(("3D printing" OR "three-dimensional printing" OR "bioprinting" OR "additive manufacturing" OR
"tissue engineering" OR "scaffold*" OR "biofabrication" OR "biomaterial*") AND TS=("auricular
reconstruction" OR "ear reconstruction" OR "microtia" OR "auricular cartilage" OR "ear cartilage" OR
"pinna" OR "auricle" OR "external ear"))
```

The publication window was 1 January 2014 to 31 December 2025. The retained document types were Article and Review, and the retained language was English. The lower date bound was selected to capture the period in which extrusion-based bioprinting and patient-specific fabrication of ear-shaped cartilage constructs became a sustained research direction. The database search and WoSCC `Cited_By_Count` snapshot were recorded on 12 June 2026; supplementary metadata enrichment was completed on 13 July 2026; and the analytical files were locked on 6 September 2026.

Selection stage	Records retained	Change from previous stage
WoSCC topic search	1542	—
After 2014–2025, Article/Review, and English limits	1253	289 excluded
After title/abstract relevance screening	569	684 excluded
After topic screening	386	183 excluded
After preliminary planning/model screening	310	76 excluded
After initial full-text assessment	294	16 excluded
Candidate records entering final eligibility audit	264	30 excluded before candidate set
Final included corpus	169	95 excluded in final audit

The complete record-level decision history is available for the 294-record late-stage set, which comprises the 264 candidate inclusions and 30 earlier documented late-stage exclusions. The earlier 1,253-record screening-pool export was not available in the retained project files; therefore, the submission reports the documented early-stage counts without claiming record-level reconstruction of unavailable early-stage decisions.

S3. Eligibility Criteria and Final Audit

Eligible empirical studies were required to fabricate, test, implant, or clinically apply an auricular-directed reconstructive scaffold, biomaterial, biofabricated construct, or tissue-engineered strategy. Reviews required a substantive synthesis of auricular engineering. Eligible topics included external ear or pinna reconstruction, microtia or anotia reconstruction, auricular cartilage regeneration or replacement, three-dimensional printing, bioprinting, additive manufacturing, biomaterials, scaffold fabrication, and related patient-specific engineering methods.

Records were excluded when they addressed developmental biology, molecular mechanisms, cell characterization, native-cartilage characterization, or biologic injection without a qualifying engineered construct. Additional exclusions comprised non-auricular targets; temporary planning, imaging, simulation, training, or anatomical models without an implanted or biologically evaluated reconstructive construct; pure prostheses or epitheses; hearing devices; radiotherapy boluses; external supports or orthoses; conventional surgery without a qualifying engineered component; in-silico designs without fabrication or biological/clinical evaluation; stakeholder or perception studies; conference abstracts; theses; correspondence; other non-full-length publication forms; retracted records; and records for which available evidence was insufficient to establish eligibility.

The final eligibility audit excluded 95 of the 264 candidate records and retained 169 publications. Each released decision includes an immutable `Record_ID`, previous status, final decision, reason family, decision rationale, confidence level, and evidence source. `Record_ID` equals `AR-` followed by the zero-padded original record number and remains stable whether a record is included or excluded.

Final-audit exclusion reason	Records
Planning, model, template, or training only	34
Insufficient direct engineering evidence	14
Cellular, molecular, or descriptive study only	10
Conference or supplement abstract	9
Prosthesis or epithesis	8
Conventional surgery without a qualifying engineered construct	6
Non-auricular target or non-auricular-specific review	3
Hearing or audiology device	3
External support or orthosis	2
Stakeholder or perception study	2
Ineligible publication form or source	2
Radiotherapy bolus	1
Retracted work	1
**Total**	**95**

S4. Data Fields, Dates, and Coverage

The final master table preserves the WoSCC publication-year field for annual analyses. Early-online dates and formal issue-assignment dates were not substituted into the annual series. Raw citations use the fixed WoSCC `Cited_By_Count` snapshot of 12 June 2026. CrossRef citation counts are retained only as auxiliary metadata and are not substituted for WoSCC values.

Field	Records	Coverage
Abstract	146	86.4%
Author keywords	91	53.8%
Affiliation strings	140	82.8%
Record-country mapping	139	82.2%
Record-institution mapping	140	82.8%
CrossRef citation field	152	89.9%

Missing values were not imputed. Every analysis reports or identifies the relevant denominator. Records lacking affiliation strings do not contribute to country or institution summaries, and records lacking author keywords do not contribute to keyword analyses.

S5. Citation-Age Sensitivity Analysis

Raw Cited_By_Count values represent a fixed 12 June 2026 snapshot. The citations-per-publication-year sensitivity indicator was calculated as:

```
CPY(i) = Cited_By_Count(i) / max(1, 2026 – publication year(i))
```

Only annual publication dates were consistently available; consequently, CPY is an annual-resolution sensitivity measure rather than exact time-normalized citation velocity. It is reported with Y-NCS and within-year percentile and is not interpreted as research quality, experimental validity, or clinical value.

S6. Structured Content Coding

The unit of coding was the individual Record_ID. Title, abstract, author keywords, and WoS document type were the only information sources. One hundred and forty-five records had substantive title-plus-abstract information. Twenty-four records required title and, when available, author-keyword information as the primary coding basis; one of these records contained an abstract but did not provide substantive coding information, explaining why Table S3 reports 146 records with an abstract. Uncertain fields were retained as NR.

Coding domain	Controlled categories or rule
Study design	Review; experimental original; clinical original; methods/modeling; other original; NR
Highest evidence stage	Review; methods/modeling only; ex vivo only; in vitro only; small-animal in vivo; large-animal in vivo; human clinical; NR
Clinical translation level	Basic/methods; in vitro proof; small animal; large animal; first-in-human/case series; comparative clinical; review not scored; NR
Construction strategy	Conventional seeded scaffold; 3D-printed acellular framework; 3D-printed cell-seeded scaffold; cell-laden bioprinting; mold/cast; decellularized native scaffold; scaffold-free; hybrid/composite; other; NR
Material family	Synthetic polymer; natural hydrogel/polymer; dECM/decellularized tissue; cellulose; silk; composite/reinforced; alloplastic clinical implant; no scaffold/cell only; other; NR
Cell strategy	Auricular/microtia chondrocytes; other chondrocytes; MSC/BMSC; ADSC; other progenitor; co-culture/mixed; cell-free/acellular; other; NR
Construct scale	Small specimen; partial auricular segment; ear-shaped construct; full-scale auricle; clinical reconstruction; NR
Scientific endpoints	Viability/proliferation; chondrogenesis/ECM; elastin/elastic cartilage; mechanics; shape fidelity; shape retention/contraction; degradation; inflammation/biocompatibility; vascularization/perfusion; integration/skin coverage; clinical/aesthetic outcome; long-term safety/adverse events
Explicit barriers	Cell supply/dedifferentiation; mechanical stability; shape retention/shrinkage; vascularization/nutrition; degradation mismatch; inflammation/immunogenicity; skin/soft-tissue coverage; manufacturing reproducibility; long-term evidence; regulatory/clinical workflow; other
Reporting elements	Sample size; follow-up duration; mechanical test; histology/matrix assay; native-cartilage comparator; adverse events

Construction, material, cell, endpoint, barrier, and reporting fields were multi-label where applicable. The highest evidence stage was assigned conservatively and had to agree with the clinical translation level. Reviews were not assigned an experimental maturity level. Endpoint and reporting fields were coded only when explicit in the title or abstract. Barriers required explicit language describing a limitation, challenge, unresolved problem, obstacle, or design target; background context alone did not qualify.

S7. Complete Main-Figure Inventory

The manuscript presents a bibliometric figure set (Figures 1–9) and a complementary translational evidence set (Figures 10–13). Exact source tables for all main figures are provided in Supplementary Figure Data, and the accompanying code archives reproduce the reported outputs.

Main figure	Content	Revision status
Figure 1	Literature selection flow	Bibliometric analysis
Figure 2	Annual output and fixed-snapshot citation profile	Bibliometric analysis
Figure 3	Country/region publication and citation profile	Bibliometric analysis
Figure 4	Institution publication and citation profile	Bibliometric analysis
Figure 5	Author publication and citation profile	Bibliometric analysis
Figure 6	Journal publication and citation profile	Bibliometric analysis
Figure 7	Author-keyword co-occurrence network	Bibliometric analysis
Figure 8	Temporal span of frequent author keywords	Bibliometric analysis
Figure 9	Descriptive author-keyword share differences	Bibliometric analysis
Figure 10	Annual CPY sensitivity analysis	Translational evidence analysis
Figure 11	Evidence stage and construction-strategy matrix	Translational evidence analysis
Figure 12	Scientific endpoints and reporting completeness	Translational evidence analysis
Figure 13	Explicit barriers and clinical translation level	Translational evidence analysis

S8. Author-Keyword Processing and Coverage

Author keywords were decoded for HTML entities and split on semicolons. Normalization comprised Unicode standardization, case folding, whitespace trimming, punctuation and hyphen standardization, exact spelling correction, singular/plural consolidation, and a limited set of unambiguous abbreviations, such as `PCL` to `polycaprolactone`. Broad topic-level semantic merging was not applied. Repeated normalized terms within a record were counted once. The complete raw-to-normalized mapping, controlled synonym table, and binary record-term incidence table are supplied in Supplementary Information 2.

Ninety-one records contributed 470 normalized record-term occurrences. Fifty terms occurred in at least two records and accounted for 255 occurrences in the thresholded analytical set. Because keyword provision varied by year and may also vary by journal indexing practice and article format, temporal keyword findings are interpreted as patterns within the keyword-bearing subset rather than complete field-wide prevalence estimates. The direction and magnitude of any resulting journal-profile bias cannot be quantified from missing author-keyword fields.

Year	Records in corpus	Records with author keywords	Records without author keywords	Coverage (%)
2014	10	4	6	40.0
2015	11	3	8	27.3
2016	12	6	6	50.0
2017	5	2	3	40.0
2018	16	7	9	43.8
2019	13	5	8	38.5
2020	14	8	6	57.1
2021	17	10	7	58.8
2022	14	8	6	57.1
2023	20	9	11	45.0
2024	17	14	3	82.4
2025	20	15	5	75.0

S9. Keyword Co-Occurrence Network

Co-occurrence was defined as the number of records in which two normalized author keywords appeared together. The complete symmetric matrix contains all 50 terms occurring in at least two records. Its diagonal equals record frequency. The displayed network retains weighted edges with co-occurrence of at least two and contains 29 non-isolated nodes and 58 weighted edges.

Network component	Specification
Input	Binary `Record_Keyword_Map`; one occurrence per normalized record-term
Node eligibility	Normalized keyword frequency of at least 2 records
Matrix	Complete 50 × 50 symmetric matrix; diagonal equals record frequency
Edge weight	Number of records containing both terms

Network component	Specification
Displayed edge threshold	Weight of at least 2
Graph	Undirected and weighted
Software	Python; NetworkX 3.6.1
Community algorithm	Louvain; co-occurrence weight; resolution 1.0; convergence threshold `1 × 10−7`; seed 42
Layout	Weighted spring layout; seed 42; 500 iterations
Output	29 non-isolated nodes; 58 edges; 5 communities
Figure encoding	Node area = record frequency; edge width = co-occurrence weight; node color = community

The complete matrix, all nonzero edges, thresholded edges, node frequencies, community assignments, degrees, weighted degrees, coordinates, plot-inclusion indicator, and record-term incidence data are supplied. Terms isolated under the edge threshold remain in the complete matrix and node table but are not displayed in Figure 7.

S10. Temporal Keyword Share-Difference Index

For each of the 50 terms with total record frequency of at least two, the descriptive index was calculated as:

```
TI(k) = n(k, 2020–2025)/336 − n(k, 2014–2019)/134
```

Here, `n(k, period)` is the number of within-record-deduplicated occurrences of term `k` in the stated period; 134 and 336 are the corresponding totals of normalized record-term occurrences. For example, `bioink = 6/336 − 0/134 = +0.017857`, `decellularization = 4/336 − 0/134 = +0.011905`, and `tissue engineering = 11/336 − 17/134 = −0.021055`. The released table provides both numerators, both denominators, period-specific shares, first and last occurrence years, and the exact index for every eligible term. The index is a descriptive keyword-share difference, not a significance test or burst statistic.

S11. Citation Normalization

Raw citation totals are fixed-snapshot descriptions. To reduce publication-age bias, each record received a within-publication-year normalized citation score:

```
Y-NCS(i) = Cited_By_Count(i) / mean Cited_By_Count of final-corpus records published in the same year
```

For an entity group, `MNCS` is the arithmetic mean of the member records' `Y-NCS` values. A tie-aware within-year percentile was calculated as:

```
Percentile(i) = 100 × [L(i) + 0.5E(i)] / N(year)
```

where `L(i)` is the number of same-year records with fewer citations, `E(i)` is the number with the same citation count, and `N(year)` is the number of final-corpus records published in that year. Zero-citation records were retained. Entity summaries also report the percentage of records at or above the 75th within-year percentile. These measures adjust only for publication year within this corpus and are not external field- or subject-category-normalized indicators.

S12. Entity Standardization and Counting

Country, institution, author, and journal outputs use whole counting. Each explicitly identified country or retained institution receives one publication credit for a multientity record. Country percentages use the 169-record corpus as the denominator and may therefore sum to more than 100%. Institutions are retained at the

explicit organization level stated in the affiliation; documented aliases are consolidated, whereas generic department or school labels are excluded. Author names are standardized case-insensitively where possible, but residual ambiguity remains when persistent identifiers are unavailable. Journal titles are standardized for spacing, case, and known title variants. Rankings are ordered by publication count, with total citations used only to break ties.
