# Supplementary material for: Three-Dimensional Printing and Tissue Engineering in Auricular Reconstruction: Global Trends, Research Frontiers, and a Translational Evidence Map
Source: Bioengineering (Basel). 2026 Sep 22;13(10):1095. doi: 10.3390/bioengineering13101095 (PMC13701565; doi:10.3390/bioengineering13101095)
Supplement: Supplementary file 1 [file bioengineering-13-01095-s001.zip › Code Reproducibility Package.zip!/combined_code_package/Full_Analysis/evidence_map/output/figures/PDF/Figure11_evidence_maturity.pdf]

Evidence maturity and technical routes in auricular reconstruction research

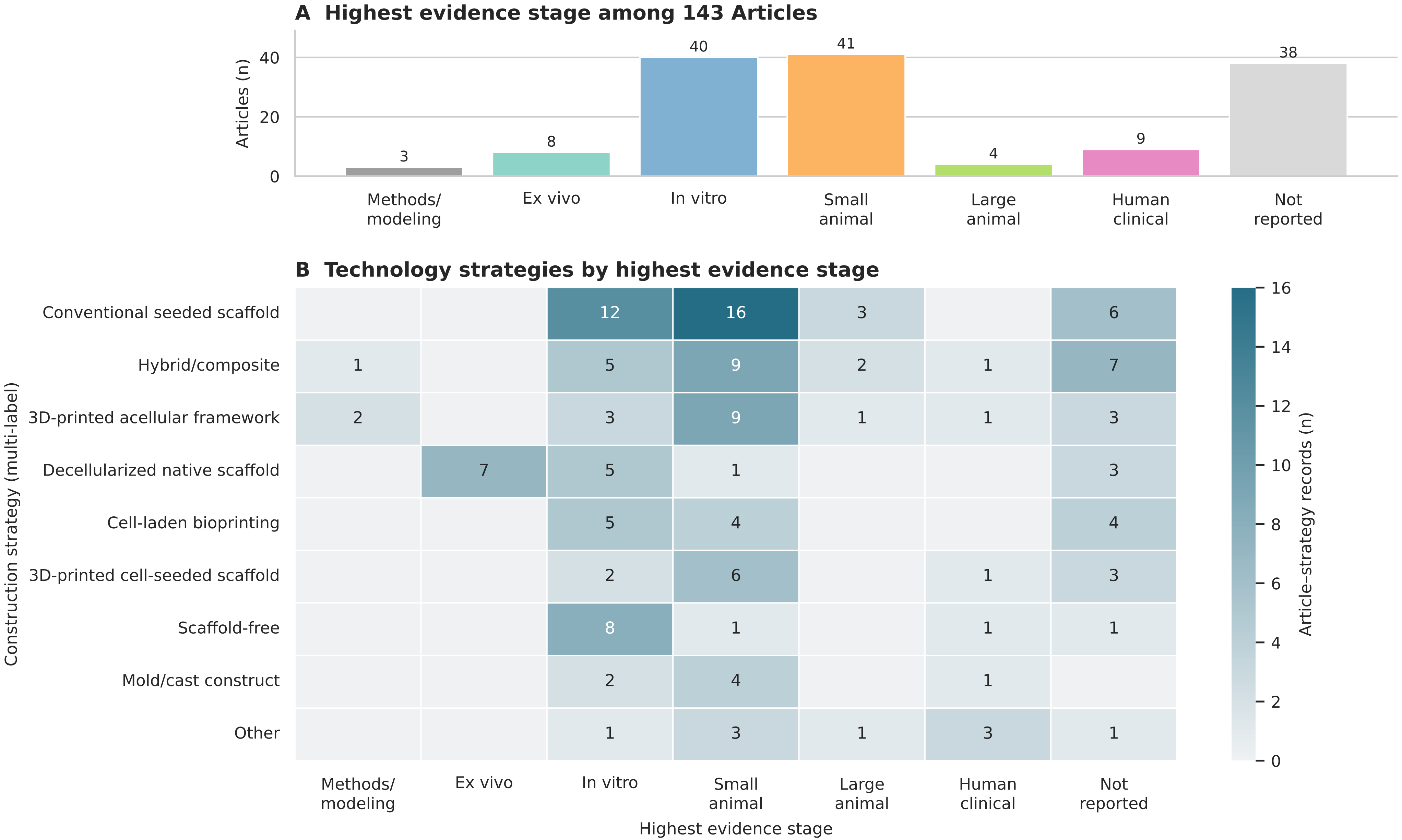

Construction strategy	Methods/ modeling	Ex vivo	In vitro	Small animal	Large animal	Human clinical	Not reported
Conventional seeded scaffold			12	16	3		6
Hybrid/composite	1		5	9	2	1	7
3D-printed acellular framework	2		3	9	1	1	3
Decellularized native scaffold		7	5	1			3
Cell-laden bioprinting			5	4			4
3D-printed cell-seeded scaffold			2	6		1	3
Scaffold-free			8	1		1	1
Mold/cast construct			2	4		1	
Other			1	3	1	3	1

Counts are based on conservative title/abstract coding. “Not reported” indicates insufficient information, not absence of experimentation.
