# Supplementary material for: Three-Dimensional Printing and Tissue Engineering in Auricular Reconstruction: Global Trends, Research Frontiers, and a Translational Evidence Map
Source: Bioengineering (Basel). 2026 Sep 22;13(10):1095. doi: 10.3390/bioengineering13101095 (PMC13701565; doi:10.3390/bioengineering13101095)
Supplement: Supplementary file 1 [file bioengineering-13-01095-s001.zip › Code Reproducibility Package.zip!/combined_code_package/Full_Analysis/evidence_map/output/figures/PDF/Figure10_citation_rate.pdf]

Citation-rate sensitivity analysis by publication year

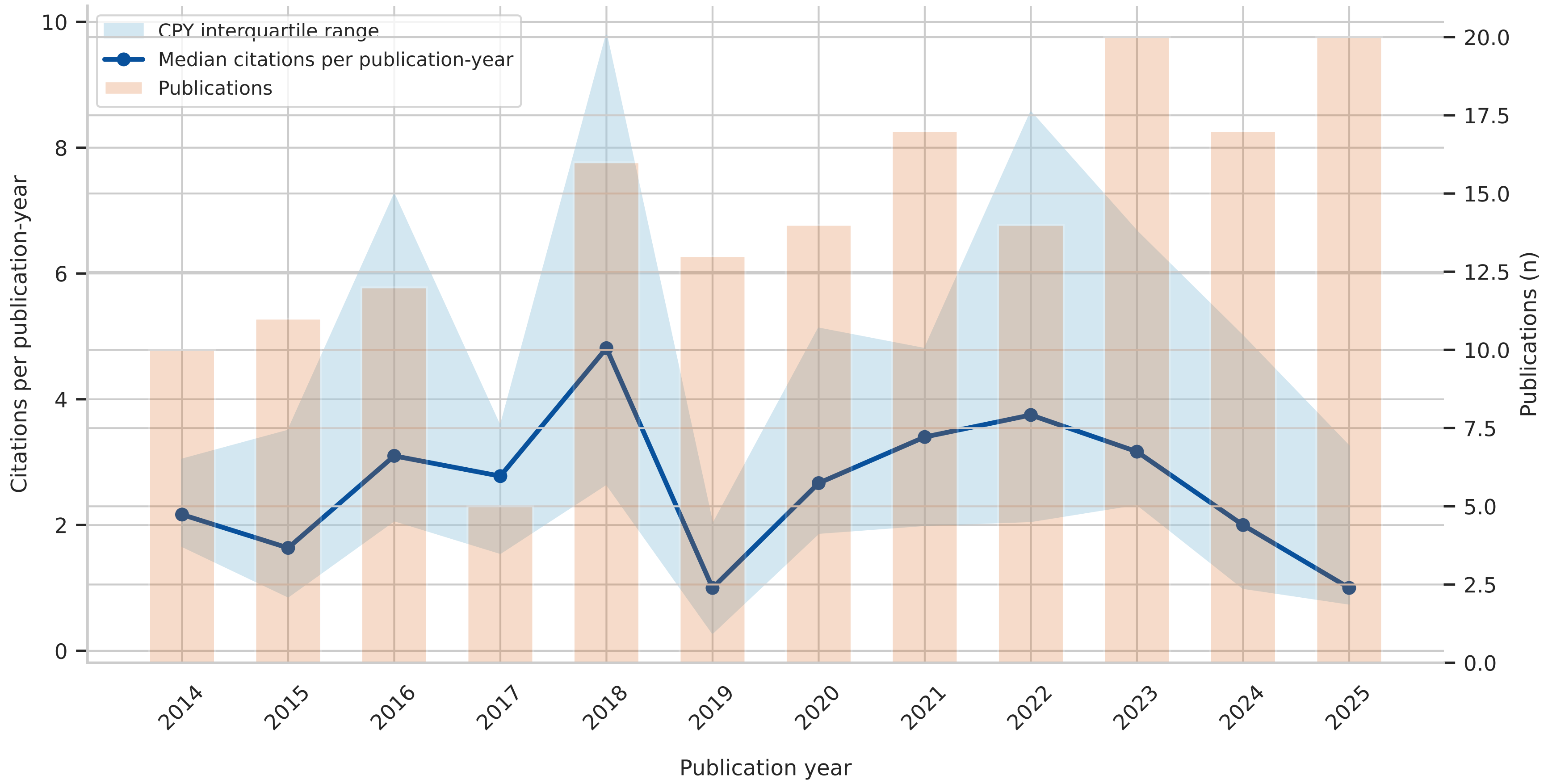

CPY = fixed WoSCC citation snapshot / max(1, 2026 − publication year). Annual resolution and partial-year exposure limit precision.
