# Supplementary material for: Three-Dimensional Printing and Tissue Engineering in Auricular Reconstruction: Global Trends, Research Frontiers, and a Translational Evidence Map
Source: Bioengineering (Basel). 2026 Sep 22;13(10):1095. doi: 10.3390/bioengineering13101095 (PMC13701565; doi:10.3390/bioengineering13101095)
Supplement: Supplementary file 1 [file bioengineering-13-01095-s001.zip › Code Reproducibility Package.zip!/combined_code_package/Full_Analysis/final/publication_figures/PDF_vector/Figure2_annual_output_citations.pdf]

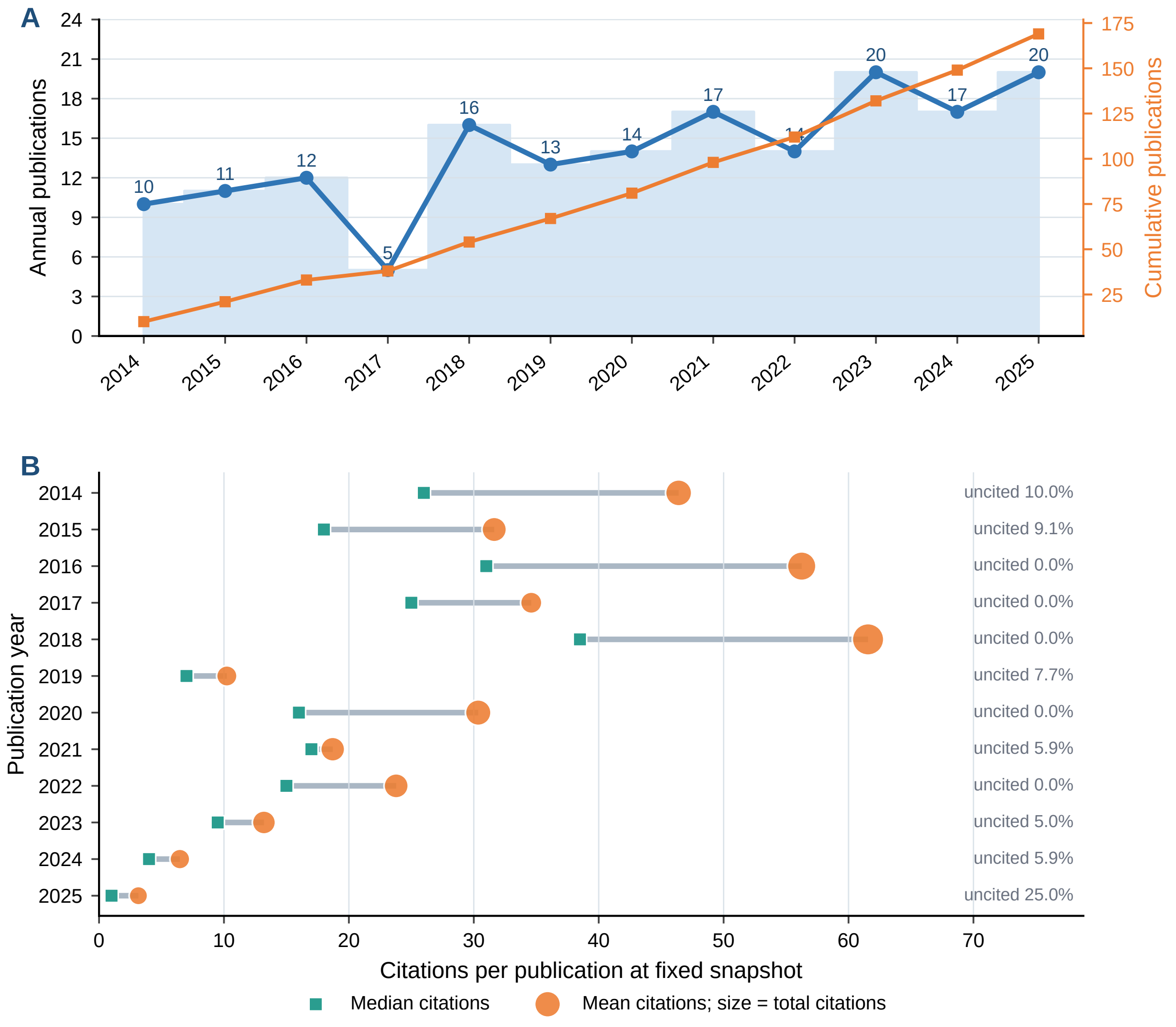
A
Annual publications
Cumulative publications
10
11
12
5
16
13
14
17
14
20
17
20
2014
2015
2016
2017
2018
2019
2020
2021
2022
2023
2024
2025
B
Publication year
uncited 10.0%
uncited 9.1%
uncited 0.0%
uncited 0.0%
uncited 0.0%
uncited 7.7%
uncited 0.0%
uncited 5.9%
uncited 0.0%
uncited 5.0%
uncited 5.9%
uncited 25.0%
Citations per publication at fixed snapshot
Median citations
Mean citations; size = total citations
