# Supplementary material for: Three-Dimensional Printing and Tissue Engineering in Auricular Reconstruction: Global Trends, Research Frontiers, and a Translational Evidence Map
Source: Bioengineering (Basel). 2026 Sep 22;13(10):1095. doi: 10.3390/bioengineering13101095 (PMC13701565; doi:10.3390/bioengineering13101095)
Supplement: Supplementary file 1 [file bioengineering-13-01095-s001.zip › Code Reproducibility Package.zip!/combined_code_package/Full_Analysis/final/publication_figures/PDF_vector/Figure3_country_analysis.pdf]

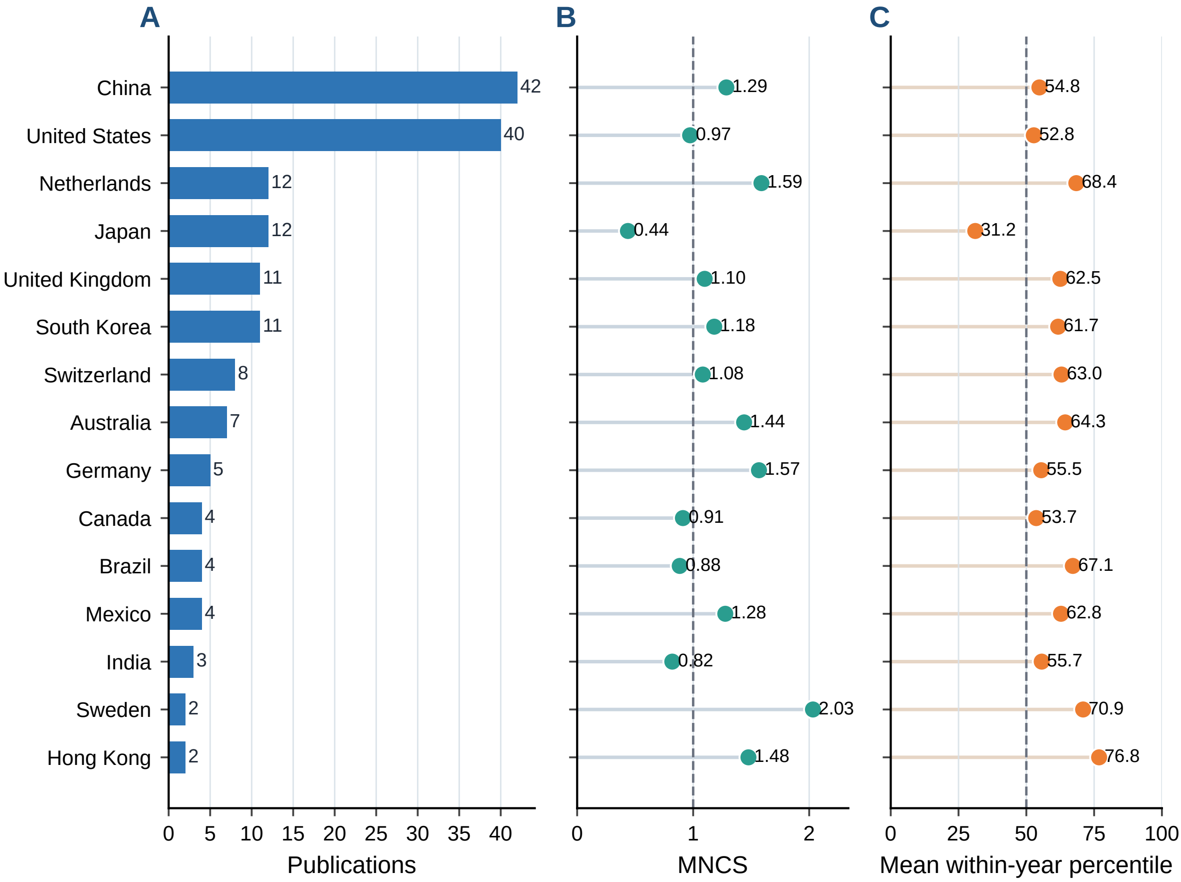

A
B
C
China
United States
Netherlands
Japan
United Kingdom
South Korea
Switzerland
Australia
Germany
Canada
Brazil
Mexico
India
Sweden
Hong Kong
42
40
12
12
11
11
8
7
5
4
4
4
3
2
2
0 5 10 15 20 25 30 35 40
Publications
1.29
0.97
1.59
0.44
1.10
1.18
1.08
1.44
1.57
0.91
0.88
1.28
0.82
2.03
1.48
0 1 2
MNCS
54.8
52.8
68.4
31.2
62.5
61.7
63.0
64.3
55.5
53.7
67.1
62.8
55.7
70.9
76.8
0 25 50 75 100
Mean within-year percentile
