# Supplementary material for: Three-Dimensional Printing and Tissue Engineering in Auricular Reconstruction: Global Trends, Research Frontiers, and a Translational Evidence Map
Source: Bioengineering (Basel). 2026 Sep 22;13(10):1095. doi: 10.3390/bioengineering13101095 (PMC13701565; doi:10.3390/bioengineering13101095)
Supplement: Supplementary file 1 [file bioengineering-13-01095-s001.zip › Code Reproducibility Package.zip!/combined_code_package/Full_Analysis/final/publication_figures/PDF_vector/Figure4_institution_analysis.pdf]

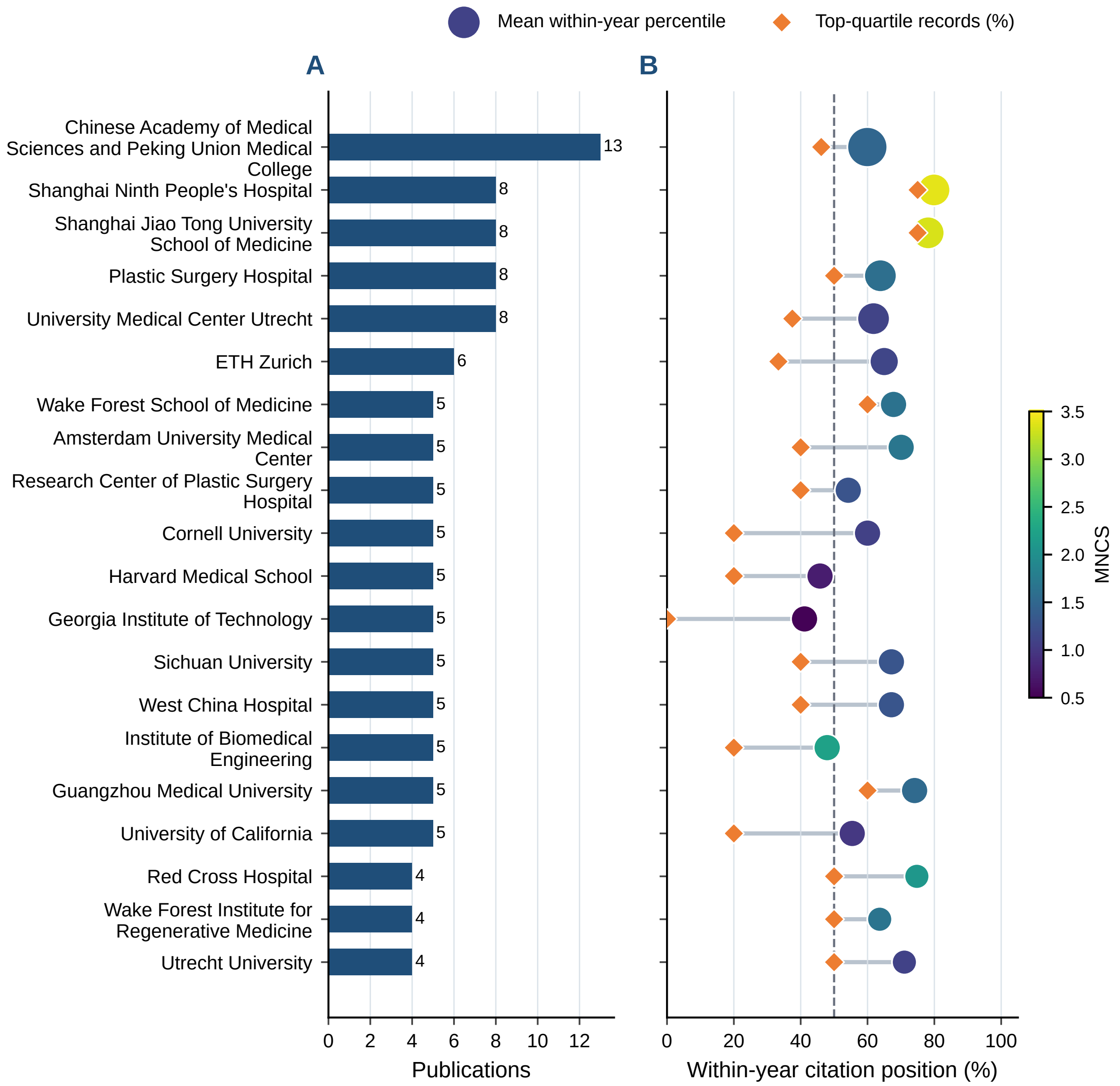

Mean within-year percentile
Top-quartile records (%)
A
B
Chinese Academy of Medical Sciences and Peking Union Medical College
Shanghai Ninth People's Hospital
Shanghai Jiao Tong University School of Medicine
Plastic Surgery Hospital
University Medical Center Utrecht
ETH Zurich
Wake Forest School of Medicine
Amsterdam University Medical Center
Research Center of Plastic Surgery Hospital
Cornell University
Harvard Medical School
Georgia Institute of Technology
Sichuan University
West China Hospital
Institute of Biomedical Engineering
Guangzhou Medical University
University of California
Red Cross Hospital
Wake Forest Institute for Regenerative Medicine
Utrecht University
13
8
8
8
8
6
5
5
5
5
5
5
5
5
5
5
5
4
4
4
0 2 4 6 8 10 12
Publications
0 20 40 60 80 100
Within-year citation position (%)
MNCS
3.5
3.0
2.5
2.0
1.5
1.0
0.5
