# Supplementary material for: Three-Dimensional Printing and Tissue Engineering in Auricular Reconstruction: Global Trends, Research Frontiers, and a Translational Evidence Map
Source: Bioengineering (Basel). 2026 Sep 22;13(10):1095. doi: 10.3390/bioengineering13101095 (PMC13701565; doi:10.3390/bioengineering13101095)
Supplement: Supplementary file 1 [file bioengineering-13-01095-s001.zip › Code Reproducibility Package.zip!/combined_code_package/Full_Analysis/final/publication_figures/PDF_vector/Figure5_author_analysis.pdf]

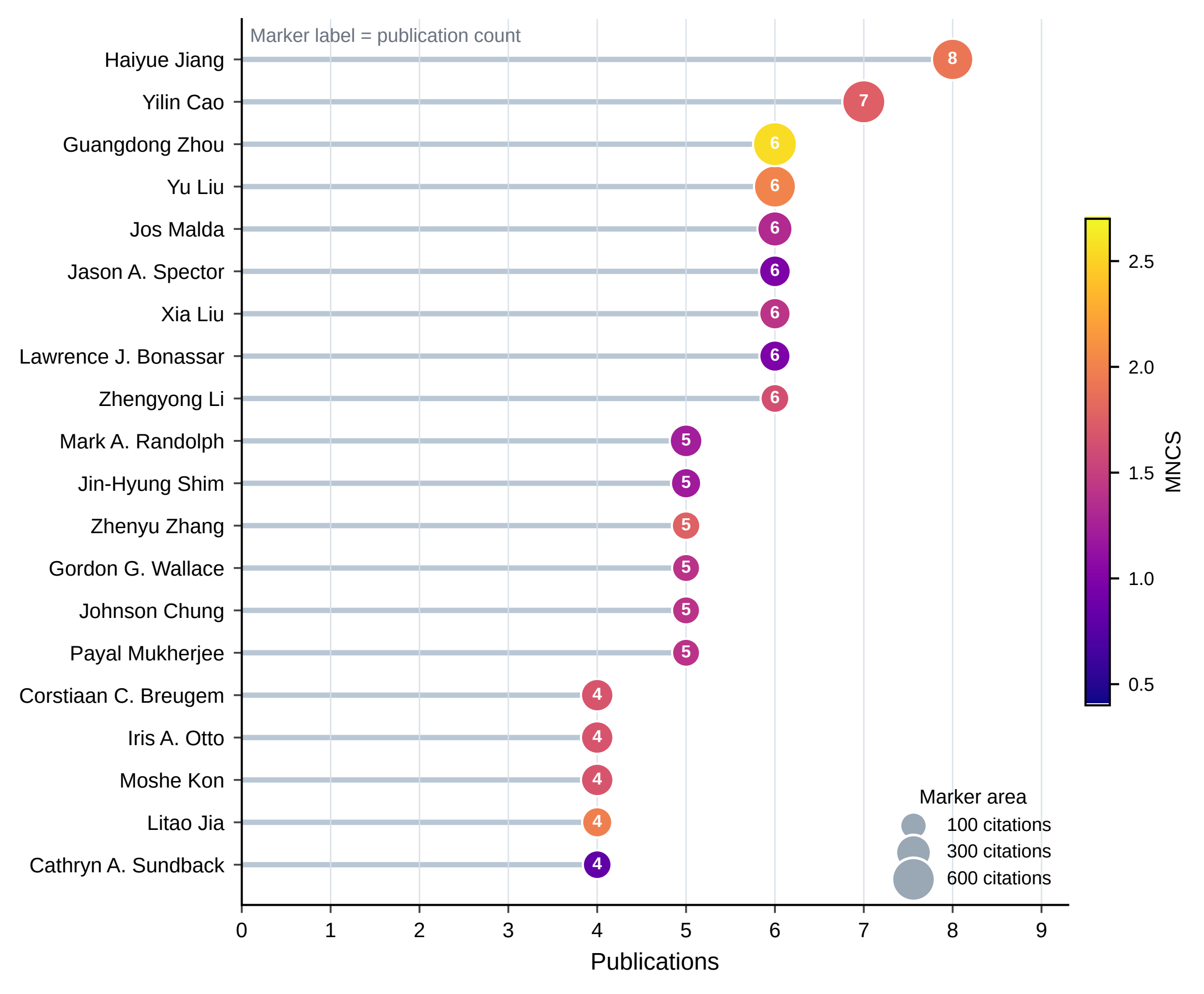
Marker label = publication count
Haiyue Jiang
Yilin Cao
Guangdong Zhou
Yu Liu
Jos Malda
Jason A. Spector
Xia Liu
Lawrence J. Bonassar
Zhengyong Li
Mark A. Randolph
Jin-Hyung Shim
Zhenyu Zhang
Gordon G. Wallace
Johnson Chung
Payal Mukherjee
Corstiaan C. Breugem
Iris A. Otto
Moshe Kon
Litao Jia
Cathryn A. Sundback
8
7
6
6
6
6
6
6
6
5
5
5
5
5
5
4
4
4
4
4
Publications
0
1
2
3
4
5
6
7
8
9
Marker area
100 citations
300 citations
600 citations
MNCS
2.5
2.0
1.5
1.0
0.5
