# Supplementary material for: Three-Dimensional Printing and Tissue Engineering in Auricular Reconstruction: Global Trends, Research Frontiers, and a Translational Evidence Map
Source: Bioengineering (Basel). 2026 Sep 22;13(10):1095. doi: 10.3390/bioengineering13101095 (PMC13701565; doi:10.3390/bioengineering13101095)
Supplement: Supplementary file 1 [file bioengineering-13-01095-s001.zip › Code Reproducibility Package.zip!/combined_code_package/Full_Analysis/final/publication_figures/PDF_vector/Figure6_journal_analysis.pdf]

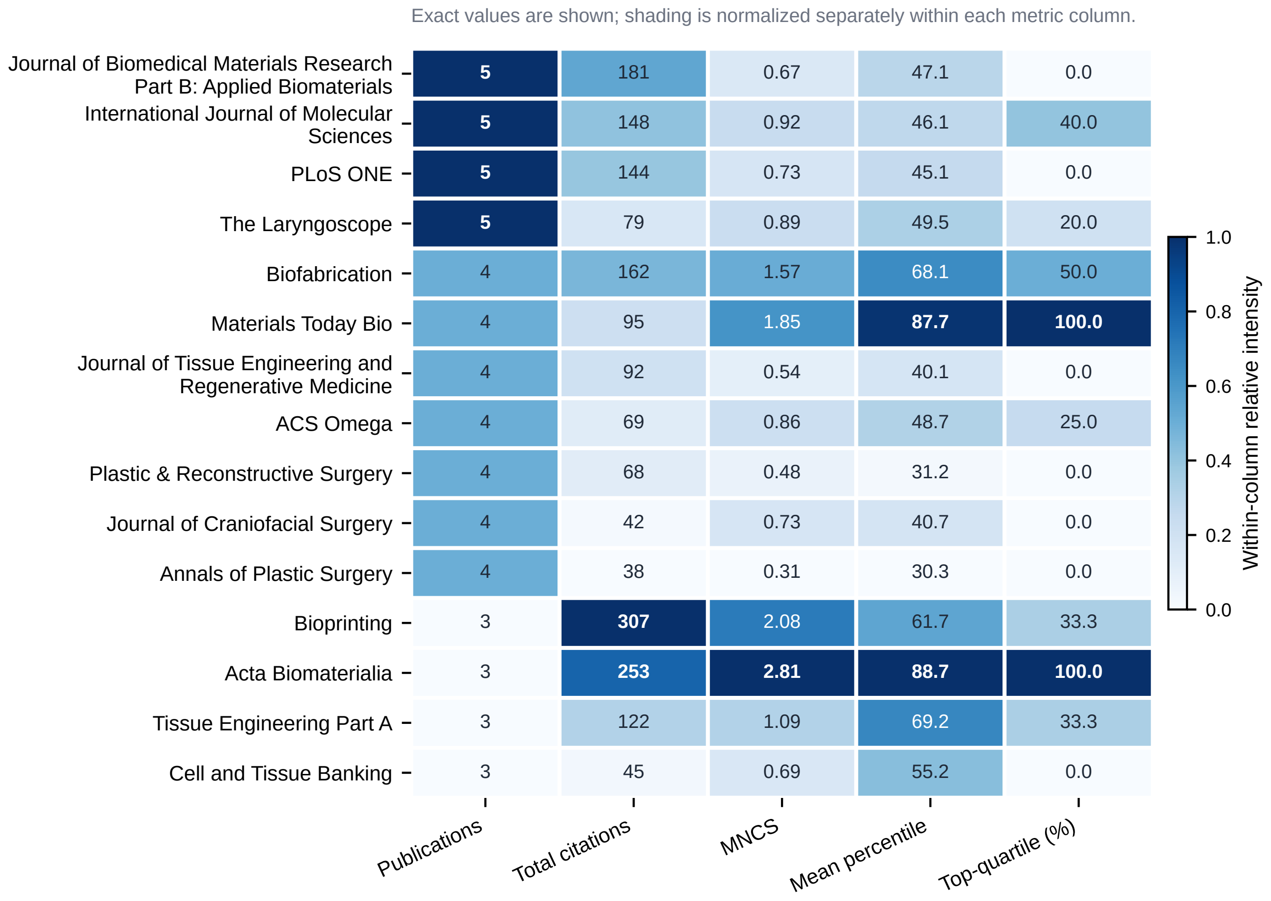

Exact values are shown; shading is normalized separately within each metric column.
Journal of Biomedical Materials Research Part B: Applied Biomaterials
International Journal of Molecular Sciences
PLoS ONE
The Laryngoscope
Biofabrication
Materials Today Bio
Journal of Tissue Engineering and Regenerative Medicine
ACS Omega
Plastic & Reconstructive Surgery
Journal of Craniofacial Surgery
Annals of Plastic Surgery
Bioprinting
Acta Biomaterialia
Tissue Engineering Part A
Cell and Tissue Banking
Publications
Total citations
MNCS
Mean percentile
Top-quartile (%)
5 181 0.67 47.1 0.0
5 148 0.92 46.1 40.0
5 144 0.73 45.1 0.0
5 79 0.89 49.5 20.0
4 162 1.57 68.1 50.0
4 95 1.85 87.7 100.0
4 92 0.54 40.1 0.0
4 69 0.86 48.7 25.0
4 68 0.48 31.2 0.0
4 42 0.73 40.7 0.0
4 38 0.31 30.3 0.0
3 307 2.08 61.7 33.3
3 253 2.81 88.7 100.0
3 122 1.09 69.2 33.3
3 45 0.69 55.2 0.0
Within-column relative intensity
0.0
0.2
0.4
0.6
0.8
1.0
