# Supplementary material for: Three-Dimensional Printing and Tissue Engineering in Auricular Reconstruction: Global Trends, Research Frontiers, and a Translational Evidence Map
Source: Bioengineering (Basel). 2026 Sep 22;13(10):1095. doi: 10.3390/bioengineering13101095 (PMC13701565; doi:10.3390/bioengineering13101095)
Supplement: Supplementary file 1 [file bioengineering-13-01095-s001.zip › Code Reproducibility Package.zip!/combined_code_package/Full_Analysis/final/publication_figures/PDF_vector/Figure8_keyword_temporal_span.pdf]

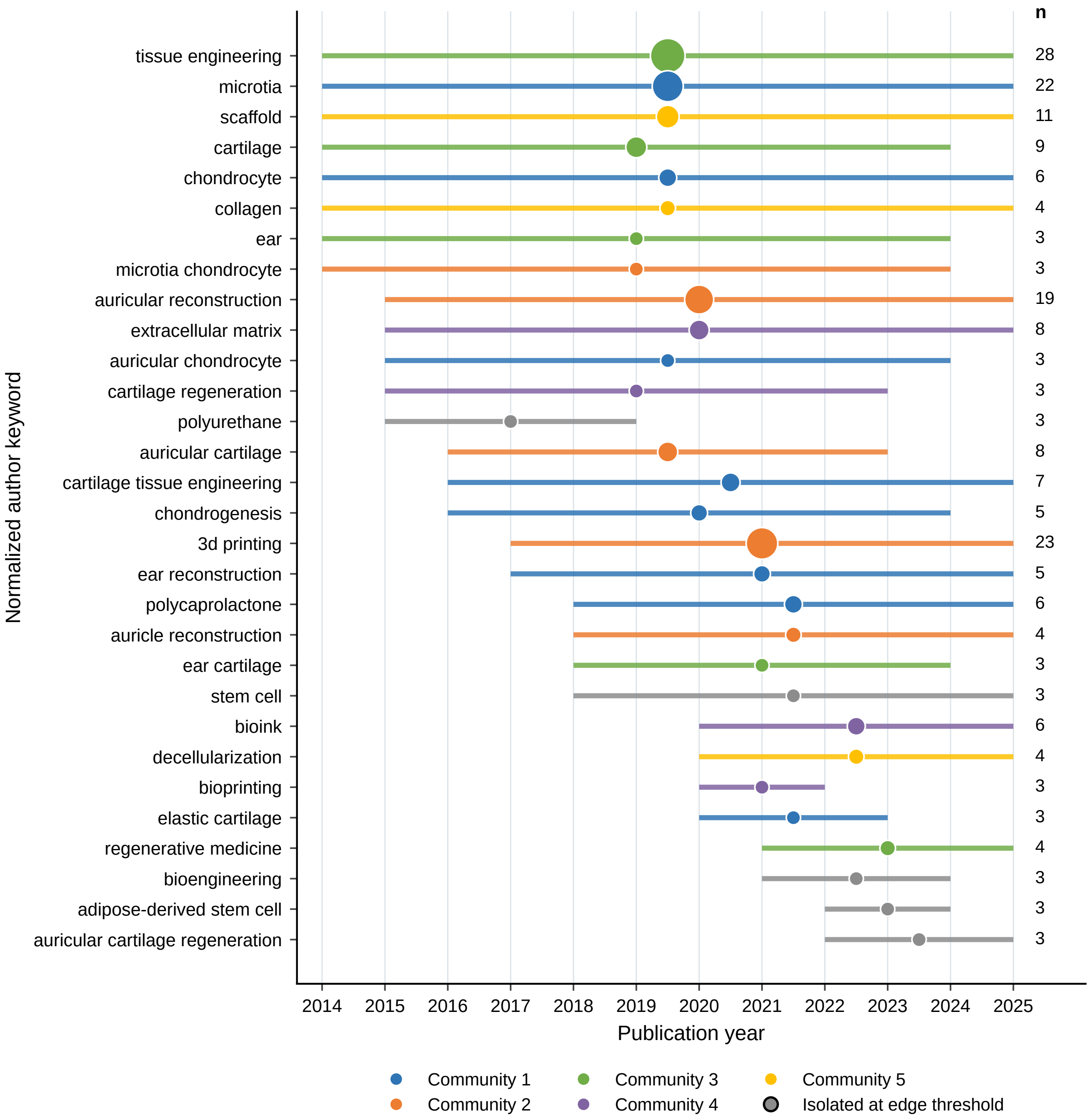

n
tissue engineering 28
microtia 22
scaffold 11
cartilage 9
chondrocyte 6
collagen 4
ear 3
microtia chondrocyte 3
auricular reconstruction 19
extracellular matrix 8
auricular chondrocyte 3
cartilage regeneration 3
polyurethane 3
auricular cartilage 8
cartilage tissue engineering 7
chondrogenesis 5
3d printing 23
ear reconstruction 5
polycaprolactone 6
auricle reconstruction 4
ear cartilage 3
stem cell 3
bioink 6
decellularization 4
bioprinting 3
elastic cartilage 3
regenerative medicine 4
bioengineering 3
adipose-derived stem cell 3
auricular cartilage regeneration 3
Normalized author keyword
2014
2015
2016
2017
2018
2019
2020
2021
2022
2023
2024
2025
Publication year
Community 1
Community 2
Community 3
Community 4
Community 5
Isolated at edge threshold
