# Supplementary material for: Three-Dimensional Printing and Tissue Engineering in Auricular Reconstruction: Global Trends, Research Frontiers, and a Translational Evidence Map
Source: Bioengineering (Basel). 2026 Sep 22;13(10):1095. doi: 10.3390/bioengineering13101095 (PMC13701565; doi:10.3390/bioengineering13101095)
Supplement: Supplementary file 1 [file bioengineering-13-01095-s001.zip › Code Reproducibility Package.zip!/combined_code_package/Full_Analysis/final/publication_figures/PDF_vector/Figure9_keyword_trend_index.pdf]

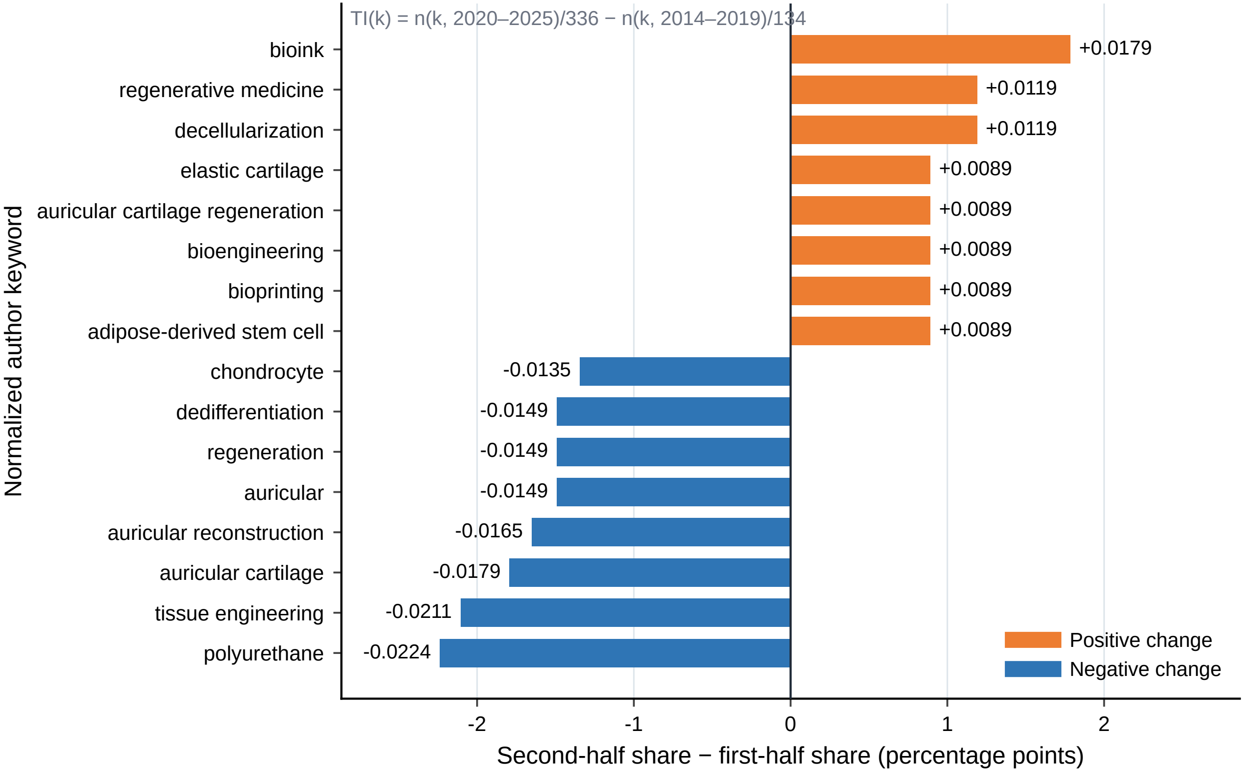
TI(k) = n(k, 2020–2025)/336 − n(k, 2014–2019)/134
bioink
regenerative medicine
decellularization
elastic cartilage
auricular cartilage regeneration
bioengineering
bioprinting
adipose-derived stem cell
chondrocyte
dedifferentiation
regeneration
auricular
auricular reconstruction
auricular cartilage
tissue engineering
polyurethane
+0.0179
+0.0119
+0.0119
+0.0089
+0.0089
+0.0089
+0.0089
+0.0089
-0.0135
-0.0149
-0.0149
-0.0149
-0.0165
-0.0179
-0.0211
-0.0224
Normalized author keyword
-2
-1
0
1
2
Second-half share − first-half share (percentage points)
Positive change
Negative change
